# Prevalence and spatiotemporal dynamics of HIV-1 Circulating Recombinant Form 03_AB (CRF03_AB) in the Former Soviet Union countries

**DOI:** 10.1371/journal.pone.0241269

**Published:** 2020-10-23

**Authors:** Aleksey Lebedev, Oksana Pasechnik, Ekaterina Ozhmegova, Anastasiia Antonova, Aleksey Blokh, Liliya Grezina, Tatiana Sandyreva, Natalia Dementeva, Elena Kazennova, Marina Bobkova

**Affiliations:** 1 Laboratory of T-Lymphotropic Viruses, Gamaleya National Research Center for Epidemiology and Microbiology, Moscow, Russia; 2 Departments of Epidemiology, Omsk State Medical University, Omsk, Russia; 3 Clinical Diagnostic Laboratory, Yamalo-Nenets Autonomous District Center for Prevention and Control of AIDS and Infectious Diseases, Noyabr'sk, Russia; 4 Clinical Diagnostic Laboratory, Sverdlovsk Regional Center for Prevention and Control of AIDS and Infectious Diseases, Ekaterinburg, Russia; 5 Clinical Diagnostic Laboratory, Saint-Petersburg Center for Prevention and Control of AIDS and Infectious Disease, Saint-Petersburg, Russia; Taif University, SAUDI ARABIA

## Abstract

**Background:**

HIV-1 circulating recombinant forms (CRFs) infections has been increasing in Former Soviet Union (FSU) countries in the recent decade. One is the CRF03_AB, which circulated in the region since late 1990s and probably became widespread in northwestern FSU countries. However, there is not much information provided about the dissemination of this recombinant. Here, we examine the prevalence, evolutionary dynamics and dispersion pattern of HIV-1 CRF03_AB recombinant.

**Methods:**

We analyzed 32 independent studies and 151 HIV-1 CRF03_AB *pol* sequences isolated from different FSU countries over a period of 22 years. Pooled prevalence was estimated using a random effects model. Bayesian coalescent-based method was used to estimate the evolutionary, phylogeographic and demographic parameters.

**Results:**

Our meta-analysis showed that the pooled prevalence of CRF03_AB infection in northwestern FSU region was 5.9% [95%CI: 4.1–7.8]. Lithuania (11.6%), Russia (5.9%) and Belarus (2.9%) were the most affected by CRF03_AB. We found that early region wide spread of HIV-1 CRF03_AB originated from one viral clade that arose in the city of Kaliningrad in 1992 [95%HPD: 1990–1995]. Fourteen migration route of this variant were found. The city of Kaliningrad is involved in most of these, confirming its leading role in CRF03_AB spread within FSU. Demographic reconstruction point to this is that CRF03_AB clade seems to have experienced an exponential growth until the mid-2000s and a decrease in recent years.

**Conclusion:**

These data provide new insights into the molecular epidemiology of CRF03_AB as well as contributing to the fundamental understanding of HIV epidemic in FSU.

## Introduction

High mutation and recombination rates of the human immunodeficiency virus type 1 (HIV-1) along with the rapid growth of its genetic diversity impede the development of an effective preventive HIV vaccine. The same properties of the virus however form the basis for studying the dynamics of evolutionary changes over time and track the patterns of virus spread during HIV-1 epidemics [1, 2]. Widespread in the last decade coalescent-based method for phylogenies inference [3] proved to be a useful tool for reconstructing the history of HIV populations in different geographical regions [4–7].

The massive economic crisis and increased drug abuse, following the collapse of the Soviet Union, have created a situation favorable for the HIV spread in the region and laying the foundation to start fastest-growing HIV/AIDS epidemic in the world [8]. To date, the Eastern Europe and Central Asia countries, formerly part of the Soviet Union (FSU), are facing an enormous challenge from the rapid spread of the HIV-1 epidemic. At the end of 2018, 1.7 million people were estimated to be living with HIV (PLWH) in this region, with an annual increase of about 160,000 in the number of new cases [8]. In spite of close economic, historical, and socio-cultural relationships between the FSU countries, the HIV-infection prevalence in the region varies from ‘low’ in Azerbaijan (0.1%) or in Lithuania (0.2%) to ‘significant’ in Ukraine (1.0%) or in Russia (1.2%) [8, 9]. Meanwhile, HIV/AIDS epidemic in FSU shares many common features (increase in drugs use and commercial sex, migration and stigmatization) including the similar molecular epidemiologic profile [10, 11].

The HIV/AIDS epidemic in FSU is driven by multiple HIV-1 M group subtypes and is far from being static. Before 1994, the number of HIV infections in FSU countries remained low, limited with nosocomial outbreaks caused by HIV-1 subtype G viruses [12], homosexual men infected with subtype B viruses [13], and heterosexual individuals (HSX) infected with multiple African clades [14]. Subsequently, HIV-1 infections began to increase dramatically due to the spread of the sub-subtype A6 strains among injective drug users (IDUs) in Odessa (Ukraine) [15–17]. Originating from a Democratic Republic of Congo (DRC) ancestor, this sub-subtype seemed to spread in Ukraine for about ten years via sexual intercourse at relatively low transmission rate before spreading explosively among IDUs (1994) [18]. After early dispersal in Ukraine, the sub-subtype A6 rapidly spread to all FSU countries along the drug trafficking routes and caused epidemics in at least twelve out of fifteen FSU countries from the Baltic to Central Asia, including Russia [19–27]. Estonia is probably the only country in FSU where non-A6 subtype (CRF06_cpx) established an epidemic [28]. Soon after that, the co-circulation of subtype A6 and non-African CRF02_AG lineage, which was first detected in Tashkent (Uzbekistan) [21] led to the generation of CRF063_02A1 which subsequently rapidly spread in Central Asian countries and thereafter moved to Russia (in 2003 and 2007) mainly by the sexual route [29, 30]. At the same time, subtype A6 generated the circulating recombinant form CRF03_AB through the recombination with subtype B clade (designated B-FSU or IDU-B) [31, 32], which led to the outbreak of HIV-infection in Kaliningrad region [33, 34]. In subsequent years, similar outbreaks were also reported in other regions of Russia [35, 36], Belarus [37] and Lithuania [26]. After expansion in northwestern FSU regions, the CRF03_AB was introduced to the Central Asian and Caucasian countries, where it was rarely detected during the last decade.

A recent meta-analysis of all published HIV-1 subtyping data in FSU countries [38] showed that subtype A6 is still responsible for almost three quarters (75.6%) of HIV-1 infections. The prevalence of HIV-1 A6 subtype is the highest, accounting for 70.0–99 90.0% of infections in Russia, Ukraine, Belarus, Latvia and Armenia [37, 39–41]. However, along with this, the prevalence of recombinants is steadily increasing. HIV-1 CRFs accounts for about 17.6% of infections with the highest prevalence in Central Asian countries, where 51.7% of all HIV-infection cases caused by recombinants are observed. During the last few years, AG recombinants (CRF02_AG/CRF63_02A1) had quickly overtaken A6 subtype in Central Asia and Russian Siberian region and to date determines the HIV-1 epidemic there (88.4% of all recombinants) [30, 38]. In contrast, the HIV-1 CRF03_AB recombinant likely represents the second most prevalent genetic form (5.2%) after subtype A6 and reaches a high prevalence (3.0%-12.0%) in Belarus, Russia, and Lithuania [26, 34, 37]. However, little is known about the evolutionary history and epidemic potential of the CRF03_AB recombinant that have expanded in the FSU countries. The objective of the present study was to summarize the literature data on HIV infection in FSU with regard to the prevalence of CRF03_AB as well as to examine the evolutionary dynamics and dispersion patterns of this recombinant in the region using the phylodynamic approaches.

## Materials and methods

### Systematic review and meta-analysis

The meta-analysis of HIV-1 CRF03_AB recombinant prevalence was conducted in Open Meta-analyst (accessible at http://www.cebm.brown.edu/openmeta/*)* using the Der Simonian & Laird method, arcs in transformation and correction factor (+0,5) for zero values as described elsewhere [38]. The heterogeneity of results between studies was evaluated using I^2^ statistic; heterogeneity was considered high at I^2^ values greater than 75.0%. Data collection was carried out in March 2020.

Briefly, we conducted a search in Russian Science Citation Index (https://www.elibrary.ru/project_rsci.asp) and PubMed (https://https.ncbi.nlm.nih.gov/pubmed/) for a depth of 22 years. Search strategy in databases: (subtype *) & (HIV) & (recombinant *) & (genotype *). Additionally, a search was carried out in Google. Scholar for the above search query, as well as manual search in bibliographic lists from the articles found. In total, 794 potentially relevant articles were identified from the search. These articles were then independently evaluated by two authors for compliance to the article review topics based on the title or abstract, duplicating etc. Published studies were enrolled if they were the following criteria: (i) articles included reports of HIV-1 subtypes in FSU; (ii) the studies included HIV-1 CRF03-AB positive people; (iii) subtyping method was clear and specific, using sequencing and phylogenetic tree analysis; (iiii) sample size of the study was more than 5 with clear and specific information for location of study (territory), material collection dates and reported prevalence of CRF03_AB. Reports of single specific subtype, duplicating publications and studies reporting conflicting data were excluded. Detailed information on the exclusion criteria is provided in S1 Fig. As a result, after removing the duplicates 637 articles remained. Of these, 535 articles were excluded after screening the titles and abstracts as not eligible, and 44 articles are not available in full texts; the full-texts were obtained for 58 articles. From these selected articles, 26 were also excluded (23 due to inconsistency with the subject of the review; 3 due to unsuitability of the data for analysis). Finally, 32 studies containing relevant data were included into the final study (S1 Table). At first, we conducted a systematic review and meta-regression analysis to CRF03_AB prevalence in Russia, for which all in-Russia studies were divided into 6 subgroups in accordance with the federal districts (Northwestern, Siberian, Far Eastern, Central, Ural and Volga). Then, we estimated the CRF03_AB prevalence in northwestern FSU countries, including northwestern federal district of Russia.

### Sequence data selection

This study was performed using the HIV-1 CRF03_AB recombinant sequences obtained from two sources. All the publicly available HIV-1 sequences from individual patients with known sampling date and location studied in the FSU countries were retrieved from the Los Alamos HIV Sequence Database (data available as of January 20, 2019; https://www.hiv.lanl.gov/). Sequences selecting was carried out manually in order to maximize the length and the number of segments for analysis. Sequences with incorrect classification were removed, resulting in a total dataset of 434 partial or complete HIV-1 CRF03_AB sequences from Azerbaijan, Belarus, Estonia, Kazakhstan, Kyrgyzstan, Latvia, Lithuania, Russia, Tajikistan, Ukraine and Uzbekistan. In four FSU countries (Armenia, Georgia, Moldova and Turkmenistan) CRF03_AB viruses were not found. Additional non-deposited sequences were collected from the Russian local AIDS centers.

After removing the duplicate sequences from the same individuals, sequences from these two sources formed the combined dataset. Having considered the number of different HIV genome regions sequences (S2 Table) from the FSU countries, we decided to focus our analysis on the HIV-1 *pol* gene sequences; this allowed maintaining a balance between (i) sequences availability for the maximum number of regional samples, and (ii) optimal length of sequences to avoid sacrificing nucleic acid substitution information. Based on these criteria, a total of 152 sequences from ten FSU countries with collection dates 1997–2019 were selected. After excluding seven low-quality sequences, only 145 sequences were subsequently used; six CRF03_AB *pol* sequences from non-FSU countries (Spain, China and United Kingdom) were additionally included (all or part) into phylodynamic and phylogeographic analyses. GenBank Accession numbers, origin information and date of HIV-1 sequences sampling used in this study are present in S3 Table. After sequence alignmenting with MAFFT [42], trimming and removing all codons associated with major drug-resistance mutations [43], the total length of the alignment was 954 nucleotides which covered the entire protease (PR) and partial reverse transcriptase (RT) regions (positions 2253–3551, HXB2-numbering).

The subtype assignment of all sequences was verified using REGA HIV subtyping tool v.3 (accessible at http://dbpartners.stanford.edu:8080/RegaSubtyping/stanford-hiv/typingtool/) and Maximum Likelihood (ML) phylogenetic analysis, as described below. For subtype verification by ML approach, pure subtype and recombinant forms reference sequences for HIV-1 group M were added to the alignment by Los Alamos HIV Sequence Database.

### Phylogenetic reconstruction and clock-likeness analysis of the phylogenies

Phylogenetic relationships among sequences were resolved using a ML tree inferred with IQ-TREE [44] under the GTR+I+G model of nucleotide substitution, which was selected as best fitting in jModelTest v2.1.7. [45]. The reliability of the phylogenies was estimated with the approximate likelihood-ratio test based on a Shimodaira–Hasegawa-like procedure (SH-aLRT). Transmission clusters were identified using ClusterPicker software with default parameters [46]. Before evolutionary and demographic reconstruction, the temporal structure of CRF03_AB *pol* datasets was estimated using TempEst v1.5 [47] by regression analysis of root-to-tip genetic distances against the sampling dates, inferred from the ML tree (S2 Fig).

### Phylodynamic and phylogeographic analysis

The rate of nucleotide substitution per site per year (s/s/y), the time to the most recent common ancestor (T_MRCA_) and the mode of population growth was jointly estimated employing the Bayesian Markov Chain Monte Carlo (MCMC) approach using the BEAST v1.10.0 [48]. We compared several molecular clock models (strict and relaxed) and coalescent priors (constant population size, exponential growth and logistic growth and Bayesian skyline) by Tracer v1.5 in order to find the best combination (S4 Table). As a result, the relaxed uncorrelated lognormal clock (UCLN) and Bayesian Skyline Plot (BSP) were used in inferring the temporal scale of the evolutionary process from the sampling dates of the sequences; a lognormal prior distribution for the clock rate were used (mean = 0.002, stdev = 0.5) [5, 49]. Two independent MCMC chains were run of 300×10^6^ steps with parameters sampled every 5000 generations, with a burn-in of first 25%.Convergence of the chains was estimated based on the Effective Sample Size (ESS) in the Tracer v1.5. The parameter estimates with ESS over 200 were accepted. Lastly, the maximum clade credibility (MCC) tree was then selected from the posterior tree distribution in TreeAnnotator v1.10.1. Final trees were visualized using FigTree v1.4.0 (accessible at http://tree.bio.ed.ac.uk/) and/or iTOL tool [50].

The estimates of ancestral geographic movements throughout the phylogenetic history were obtained using the discrete phylogeographic approach and Bayesian Stochastic Search Variable Selection (BSSVS) procedure with a CTMC rate reference prior was carried in BEAST v1.8.2. Two runs were run of 500×10^6^ steps for MCMC chains with parameters sampled every 5000 generations, with a burn-in of first 35%. The ESS value for each parameter was over 200. The MCC trees were summarized using TreeAnnotator v1.8.2 and visualized in FigTree v1.4.0. The migration routes and spatial projections were summarized using the SPREAD software [51]. The statistical support for migration routes was determined with Bayes Factor (BF); a route with the BF-support ≥ 3 was considered credible.

### Statistical analysis

Comparisons of the cluster distribution in different *FSU* countries and transmission route were performed with the Pearson chi-square test (χ2) or Fisher’s two-tailed exact test (where necessary) by using STATISTICA v.10.0 software (StatSoft, USA). The differences were considered significant at *P* less than 0.05.

## Result

### Prevalence and distribution of HIV-1 CRF03_AB recombinant in the former Soviet Union

To estimate the prevalence of CRF03_AB recombinant in FSU, we found 32 independent studies (S1 Table) that contained the relevant data on 6285 successfully genotyped samples from 6 different FSU countries (Russia, Belarus, Ukraine, Latvia, Lithuania and Estonia). Of these, CRF03_AB strains were detected in 170 cases, including 146 cases among Russian samples. The sample size of these studies varied from 7 to 1055 with a median of 141.9 patients.

Our meta-analysis revealed that the pooled prevalence of CRF03_AB in Russia was 5,9% (95% CI: 4.1–7.8) and vary among the different regions (S3 Fig). The overall proportions were as follows: 0.2% (95% CI: 0,0–0,5) for Central Federal District, 0.8% (95% CI: 0.1–1.6) for Far Eastern Federal District, 1.3% (95% CI: 0,1–2,7) for Volga Federal District, 4.6% (95% CI: 0.0–9.7) for Ural Federal District, and 17.2% (95% CI: 7.9–26.5) for Northwestern Federal District. Although no case of CRF03_AB infection was found in the Siberian federal district, this recombinant may nonetheless be present here within the confidence interval indicator of 0.3%(95% CI: 0.0–0.6). The heterogeneity was not found to be statistically significant in all Federal Districts (*P*>0.05) except the Northwestern Federal District (*P*<0.01); I^2^ ranging from 0% to 99.4%. Meta-regression analysis suggested that the CRF03_AB prevalence rate in Russia is in a downward trend (β = −0,016, *P* = 0.004) (S3 Fig).

**Fig 1:**
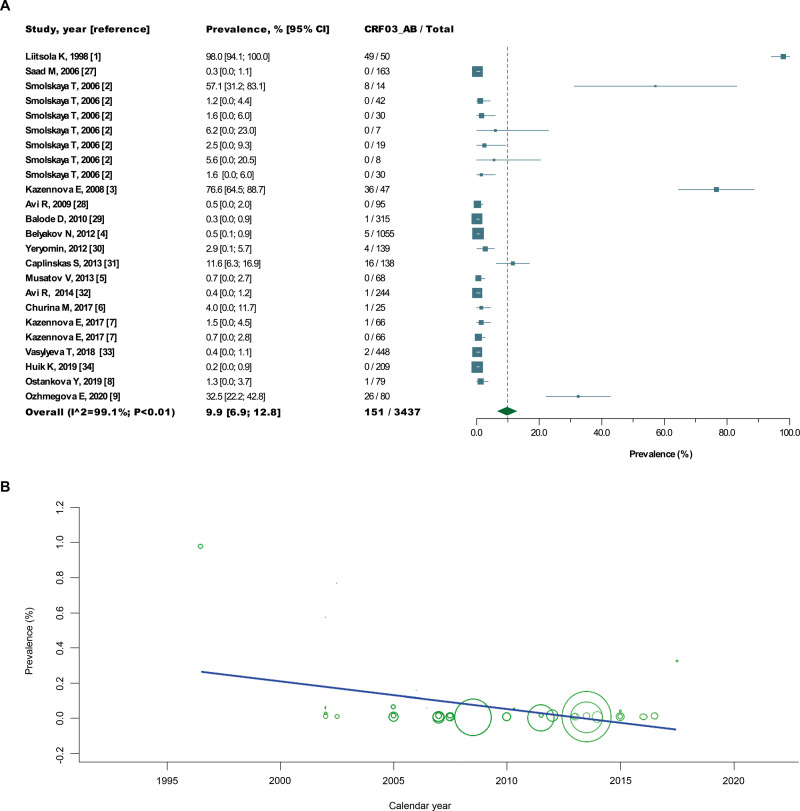
The prevalence of HIV-1 CRF03_AB recombinant in the northwestern FSU countries, including the Northwestern Federal District of Russia. The source links for forest plot (A) numbered according to the list in S1 Table. The x-axis of a meta-regression plot (B) represents year of the samples collection. The diameter of each bubble is proportional to the sample size of each study.

The pooled prevalence of CRF03_AB among different FSU countries has also varied. We estimated that CRF03_AB has a lower prevalence in Latvia (0.3%), Estonia (0.4%) and Ukraine (0.4%) than in Belarus (2.9%), with the highest prevalence in Lithuania (11.6%). In total, the pooled prevalence of CRF03_AB in these FSU countries, together with the Northwestern Federal District of Russia, was 9.9% being characterized with high heterogeneity (Fig 1A). Regarding the dynamics, the downward trend of the CRF03_AB prevalence (meta-regression; *β* = −0.013) found was not statistically significant (*P* = 0.104), which pointed to the stable circulation of this recombinant in FSU (Fig 1B).

### Origin and phylogenetic clustering of HIV-1 CRF03_AB recombinant

Here, we used the dataset consisting of 151 HIV-1 CRF03_AB recombinant *pol* sequences in total collected in FSU (*N* = 145) and other countries (*N* = 6) during 22 years (Table 1); nine out of fifteen FSU countries were covered. Among FSU sequences, 57.9% (*N* = 84) were retrieved from HIV LANL and the other 42.1% (*N* = 61) were obtained from local AIDS centers. The majority sequences were obtained in Russia (84.1%) and Lithuania (4.8%) and the remaining countries were represented by ≤5 sequences each.

**Table 1:** HIV-1 CRF03_AB recombinant *pol* sequences dataset used in this study.

Region	Country	Location ^*a*^	*N* (%) ^*b*^	Date range	Transmission route, *N* (%)					HIV prevalence (%) ^*c*^
					HSX	MSM	IDU	MTC	UNK	
**Former Soviet Union**	Azerbaijan	AZ	1 (100.0)	2002					1 (100)	0.1
	Belarus	BY	5 (22)	2000, 2008–2015					5 (100)	0.5
	Estonia	EE	1 (20)	2010	1 (100)					0.9
	Kazakhstan	KZ	3 (100)	2009–2012			1 (33)		2 (67)	0.2
	Kyrgyzstan	KG	1 (100)	2008	1 (100)					0.2
	Lithuania	LT	7 (7)	2008	1 (14)		6 (86)			0.2
	Russia incl.	RU	121 (39 ^*d*^)	1997–2019	54 (44)	1 (1)	39 (32)	1 (1)	27 (22)	1.2
	* Kaliningrad*	*Kal*	*8*	1998, 2011,2016			6 (75)		2 (25)	
	* Ekaterinburg*	*Eka*	*41*	2016–2019	22 (54)		8 (20)	1 (2)	10 (24)	
	* Cherepovets*	*Chr*	*26*	2001, 2017–2018	13 (50)		12 (46)		1 (4)	
	* Saint-Petersburg*	*Spb*	*8*	2007–2014, 2018	5 (62)	1 (13)	2 (25)			
	* YaNAD*	*Yam*	*13*	2004–2013	10 (77)		2 (15)		1 (8)	
	* Krasnodar*	*Krd*	*3*	2008, 2015–2016	2 (67)				1 (33)	
	* Tyumen’*	*Tum*	*4*	2006, 2015–2016			2 (50)		2 (50)	
	* Perm’*	*Prm*	*4*	1998–1999, 2011	1 (25)		3 (75)			
	* Others*	*Oth*	*14*	2008–2016	1 (7)		4 (29)		9 (64)	
	Tajikistan	TJ	5 (100)	2017					5 (100)	0.2
	Ukraine	UA	1 (17)	2015	1 (100)					1.0
	**All**		**145 (000)**	**1997–2019**	**58 (40)**	**1 (1)**	**46 (31)**	**1 (1)**	**39 (27)**	
**Others**	Spain	ES	4 (57)	2007, 2012					4 (100)	0.3
	China	CN	1 (100)	2013			1 (100)			N/a
	United Kingdom	UK	1 (100)	2013	1 (100)					0.2
	**All**		**6 (67)**	**2007, 2012–2013**	**1 (17)**		**1 (17)**		**4 (66)**	

^a^ Location assigned in the phylo(-geographic)genetic analysis.

^b^ Number of partial HIV-1 *pol* sequences for each country (percentage of total number of HIV-1 CRF03_AB sequences for country; only one sequence per patient was considered).

^c^ HIV prevalence data indicated according to the UNAIDS report and National report on the Russian Epidemic.

^d^ Taking into account the sequences obtained in this study. Abbreviation: HSX, heterosexual contacts; IDU, injection drug users; MSM, men who have sex with men; MTC, mother-to-child transmission; UNK, unknown; YaNAD, Yamalo-Nenets Autonomous district; N/a, not available.

**Fig 2:**
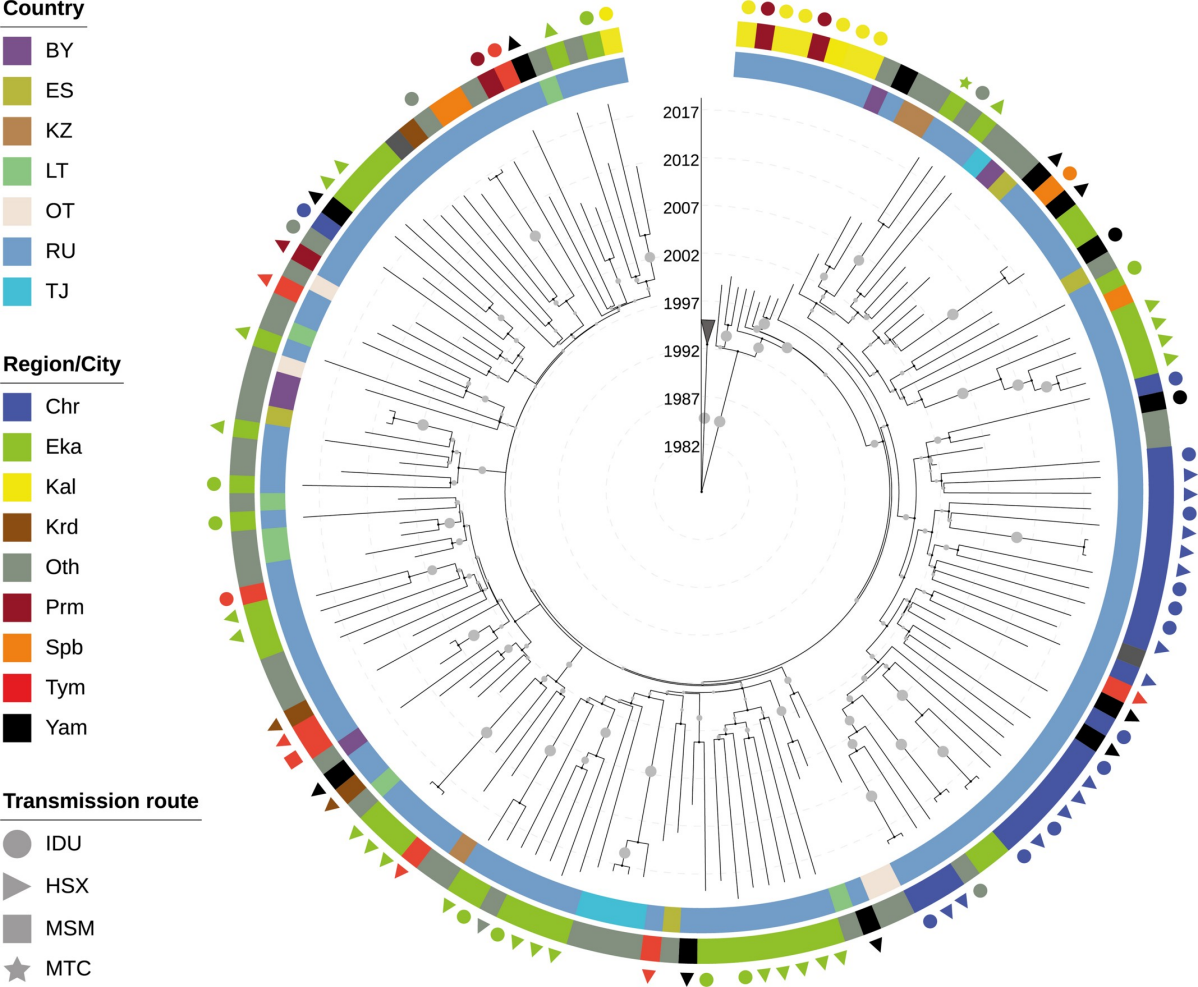
Bayesian time-scaled maximum clade credibility tree of 151 HIV-1 CRF03_AB recombinant *pol* sequences from former Soviet Union and neighboring states. Tree was rooted using FSU HIV-1 A6 sequences as outgroup (presented in collapsed form). The branch support (Posterior probability) is mapped with the circles using a grey color gradient/size (greater value, darker grey/greater circle). Concentric circles show the geographical regions of origin patient, symbols indicate the transmission route. Countries and separate cities (regions) are mapped on the inner and external concentric circle, receptively, using a color code as indicated in the legend. Branch lengths of Bayesian tree are drawn to scale with the concentric circles indicating calendar years. Abbreviation: BY, Belarus; ES, Spain; KZ, Kazakhstan; LT, Lithuania; OT, Other (countries with 2 or less available sequences; Table 1); RU, Russia; TJ, Tajikistan; Chr, Cherepovets; Eka, Ekaterinburg; Kal, Kaliningrad; Krs, Krasnodar; Oth, Other (cities with 2 or less available sequences; Table 1); Prm, Perm’; Spb, Saint-Petersburg; Tym, Tyumen’; Yam, Yamalo-Nenets Autonomous district; HSX, heterosexual contacts; IDU, injection drug users; MSM, men who have sex with men; MTC, mother-to-child transmission.

The Maximum-likelihood and Bayesian phylogenetic approaches showed that the CRF03_AB sequences were combined in one well supported monophyletic clade (Posterior Probability, *PP* = 1.0), confirming the common ancestry of each sequence (Fig 2 and S4 Fig). According to the Bayesian evolutionary analysis under a Bayesian Skyline prior, the mean evolutionary rate [95% HPD] of the PR-RT genomic region was 2.46x10^-3^s/s/y [1.74–3.18x10^-3^]. Considering these substitution rates, the MRCA for CRF03_AB clade was estimated to be 1992.1 [95% HPD: 1990.0–1995.2] (Fig 2); the root location of ancestor for this clade was traced with most probability to Kaliningrad (Posterior State Probability, *PSP* = 0.97) (S4 Fig). Despite the high degree of the intermixing of CRF03_AB sequences from FSU countries/group, the phylogenetic ML analysis revealed the existence of 11 transmission clusters (S5 Fig). In total, 25 sequences (16.5%) from the dataset were included in these clusters. However, these clusters containing 2 to 4 patients, showed short branches in the ML tree, which pointed to the short time frame between infections, and had common geographic origin; for this reason, they were considered to be of little interest. Among the cluster sequences that could be classified by the transmission route, the most prevalent were the HSX (44,4%; *P* = 0.490 *vs* IDU).

### Migration pathway of the HIV-1 CRF03_AB recombinant

**Fig 3:**
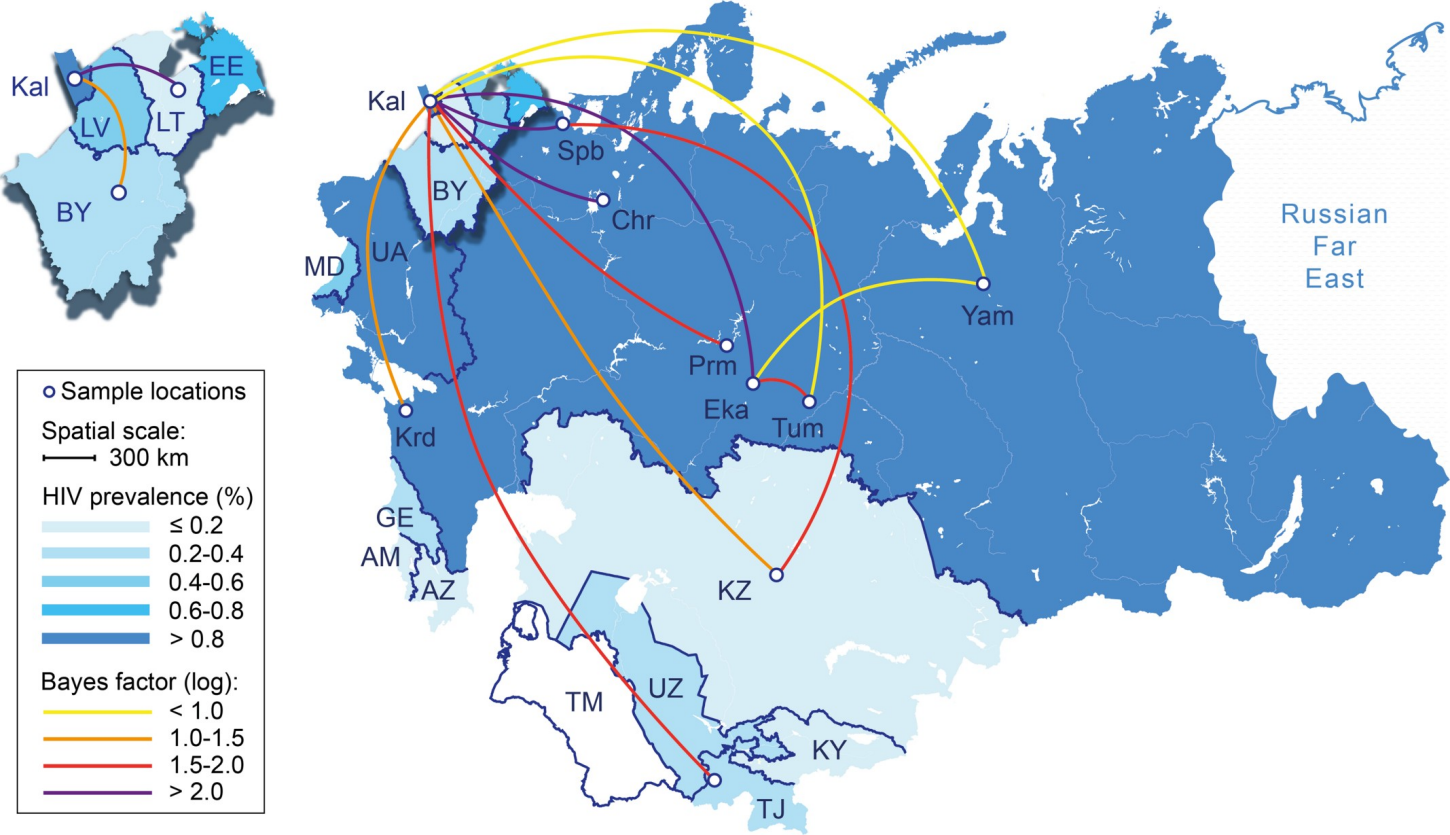
The migration patterns of the HIV-1 CRF03_AB recombinant in the former Soviet Union. The geographic locations are colored according the HIV-1 prevalence data [8]. Lines between the locations indicate the migration routes between the CRF03_AB sub-populations and correspond to the location state transitions along the branches of the Bayesian MCC tree. Line colors reflect the Bayes Factor test support for epidemiological linkage between locations. Location abbreviations are presented in Table 1 or Fig 2. Republished from [52] under a CC BY license, with permission from Hellerick, original copyright 2014.

Phylogeographic analysis was performed using only countries/locations with at least 3 available sequences, resulting in a dataset of 137 sequences from six countries (Russia, Belarus, Lithuania, Kazakhstan, Tajikistan and Spain); Russian sequences were represented by seven separate regions/cities (Table 1). The estimation of genetic flow between different locations supported at least 14 HIV-1 CRF03_AB recombinant migrations events, eleven of which involved the city of Kaliningrad (Fig 3). In addition, the root of the phylogeographic tree, as mentioned above, was located in this city. The most strongly supported viral migration route was found between Kaliningrad and Ekaterinburg (logBF = 4.9), Kaliningrad and Cherepovets (logBF = 3.9), and Kaliningrad and Saint-Petersburg (logBF = 3.7). Five other routes connecting Kaliningradto Kazakhstan, Tajikistan, Belarus, Lithuania, and Spain were slightly less supported (2.0>logBF>1.0). Finally, the reliable genetic flow (logBF≥0.5) was also found between Kaliningrad and Perm’, Ekaterinburg, Tumen’, Krasnodar, and Yamal. Three remaining routes connected Saint-Petersburg to Kazakhstan, Ekaterinburg to Tumen, and Ekaterinburg to Yamalo-Nenets Autonomous district (Fig 3).

### Demographic history of HIV-1 recombinant CRF03_AB in former Soviet Union

**Fig 4:**
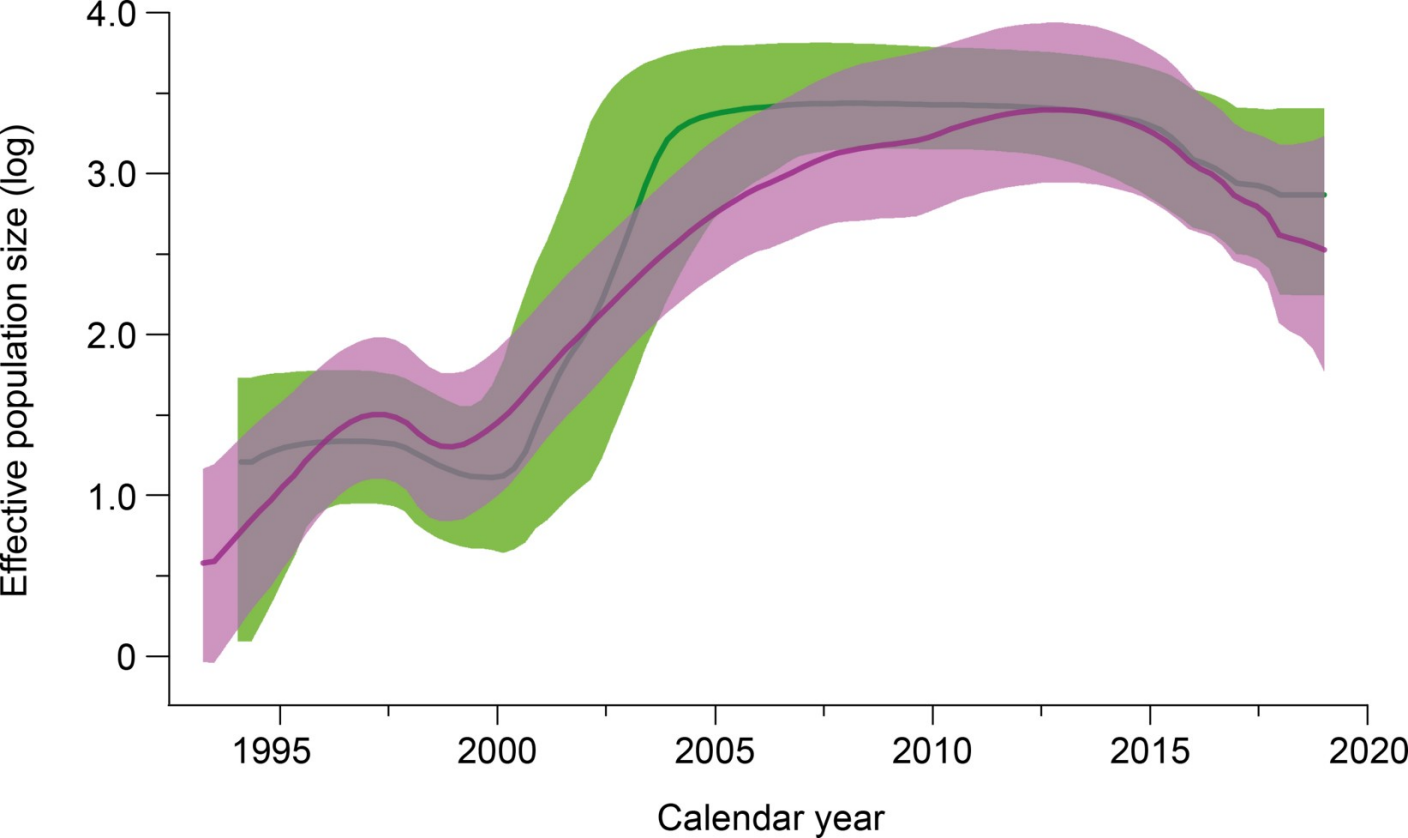
Demographic history of the HIV-1 CRF03_AB recombinant in the former Soviet Union. Estimates of the effective population size (Ne) through time (t) using Bayesian Skyline (green) and Bayesian GMRF (violet) coalescent model are presented on a logarithmic scale as median (solid color line) with the corresponding 95% HPD credibility interval (color area).

All 145 available CRF03_AB sequences from FSU were used to reconstruct the demographic history of this HIV-1 recombinant in the region (Fig 4). The reconstruction of population dynamics with the Bayesian Skyline model suggested that the CRF03_AB recombinant in FSU experienced an initial rapid growth until the beginning of the 2004, it reached a stabilization of the effective population size in 2006, and then declined after 2015. It is notable that the Bayesian (GMRF) Skyrid model, supported a longer growth phase until late 2010, followed by a population size decline that extended till present time; though this model may uncover some aspects of the population history undetected by other Bayesian models [53], we rely on the BSP data here.

## Discussion

The prevalence of different HIV-1 subtypes have experienced a tremendous change in the FSU region and the present epidemic here is predominantly driven by heterosexual practice [8]. In recent decade, the epidemic evolved with an increase both in the incidence and prevalence of HIV-1 non-A6 subtypes, primarily recombinant forms [38]. Hence, recombinant forms spread are becoming a serious public health challenge, which requires a deeper understanding of their origin and distribution. Among the recombinant forms in FSU, HIV-1 CRF03_AB recombinant is one of the common recombinants and even the second most prevalent HIV-1 subtype [26] in some countries (Lithuania). We report here the results of the first systematic review of the CRF03_AB infection prevalence based on 32 eligible studies and also present its spatiotemporal dynamics in the FSU using publicly available sequences and Bayesian phylogenetic approach.

This review showed that the pooled prevalence of HIV-1 CRF03_AB recombinant in northwestern FSU countries, including the Northwestern federal district of Russia, accounts to nearly about a tenth of all HIV-infections (9.9%), with clear geographical differences between the countries; this figure is higher than our previous estimate of about 5.2% [38]. Nonetheless, compared to that study (only partially overlapping with the present one), this analysis incorporated more eligible articles, including all studies in the systematic review [38] as well as additional studies published from 1998 to 2010 and 2018 to 2020; hence, up-to-date information and extended list of studies is critical.

The result of our study showed that the countries to which the recombinant virus first entered and established an epidemic–Lithuania (11.6%), Russia (5.9%) and Belarus (2.9%) were the most affected by CRF03_AB infection, while in neighboring Baltic FSU countries–Estonia (0.4%) and Latvia (0.3%), as well as Ukraine (0.3%), this recombinant displayed a much more restricted local dissemination. This indicates that the extraordinary epidemic consequence of HIV-1 CRF03_AB recombinant is probably associated not only introduction into highly connected transmission networks, but also with an earlier entry into this group. Although the prevalence of CRF03_AB infection is in a stable (low) trend, this recombinant is the third most prevalent HIV-1 CRF in northwestern FSU countries. Unfortunately, the scarcity of data regarding the risk factors for HIV infection in the dataset did not enable comparison of estimated prevalence between different populations. In Russia, the Northwestern federal district (17.2%), Ural federal district (4.6), and Volga federal district (1.3%) had a high-level epidemic of HIV-1 CRF03_AB recombinant. Taken together, these results seem to indicate that CRF03_AB has largely contributed to the HIV epidemic in the region and may affect the epidemic in the future.

Nevertheless, given the high heterogeneity between studies, the pooled prevalence estimates should therefore be interpreted with caution. In particular, the heterogeneity in pooled prevalence estimates may be due to the bias introduced by study methods, geographical location of the study, sample size, and year of data collection, or data collection method. It is not excluded that this latter factor is a major source of heterogeneity in CRF03_AB prevalence studies in FSU as the majority of included studies apparently were based on "convenience sampling".

The previous molecular epidemiological studies [33, 34] suggest that CRF03_AB arose in Kaliningrad region, earlier than in any other FSU regions. Here, we tested this hypothesis using the sequence dataset of twelve FSU locations, including the samples described by Liitsola et al. (1998) [33].

In the mid-1990s, Kaliningrad, an enclave of Russian territory at the Baltic Sea between Lithuania and Poland was experiencing an explosive HIV-infection epidemic, which affected IDUs and their sexual partners. Only between June 1996 and April 1997 more than 1200 HIV cases were detected in Kaliningrad population of about half a million inhabitants [54]. Like in other IDU-associated HIV-infection epidemics in FSU at that time, the main mode of HIV transmission was syringe and needle-sharing but unlike them in Kaliningrad, the non-A6 subtype (CRF03_AB) established an epidemic. Our study suggests that the CRF03_AB clade originated in Kaliningrad region approximately in 1992 and showed a rapid initial growth from the late 1990s. Although the drug use has since then slowed the number of drug-related HIV infections in FSU increased since the late 1990s and have increased further since the beginning of 2000s [55–57]. These historical records support our phylodynamic results and may explain the rapid initial dissemination of CRF03_AB by the introduction in this highly connected network. It should be noted that this demographic model appears to be similar to the A6 sub-subtype epidemic in the former Soviet Union, where injecting drug use was a key factor in determining the pattern of HIV epidemic growth. In comparison, «A6-like» epidemics also seem to have experienced a rapid expansion over the first years, followed by a decline in growth rate. Since then, the transmission patterns in FSU have gradually evolved from injecting drug use to heterosexual contacts [8]. In combination with the saturation of high-risk transmission network and the access to highly active antiretroviral therapy started in region this in mid-2000, this may have contributed to the reduction of the effective HIV-1population size [58]. However, although heterosexuals are making a greater contribution to the epidemic now, the cross-risk group transmission via IDUs is still relevant for CRF03_AB (Table 1); this partially explains why no clusters were detected among the high-risk groups. The phylogenetic analysis revealed the high level of CRF03_AB sequences intermixing from FSU countries, which provides the evidence against the existence of country-specific lineages. It may be possible that such a low level of geographic compartmentalization of the HIV-1 epidemic in FSU is associated with the continuous cross-border virus transfer between countries due to historical ties and intense human mobility.

The phylogeographic reconstruction carried out in the present study supports the idea that the CRF03_AB clade could have been initially originated in Kaliningrad, with further spreading to the West (Lithuania, Belarus) and North (Saint-Petersburg) through short-distance transfers. The epidemiological links between these locations are not unexpected due to their geographical proximity and high level of people migration, which was constant at the end of the 20th century even after the USSR collapse. Although this results pointed to the Kaliningrad as the geographical origin of CRF03_AB, this clade might have arose elsewhere since no parental subtype A or subtype B strains have been reported in Kaliningrad [33]. Probably as previously reported [32, 33], the original recombination event took place in Ukraine, where parental forms were found, and then the virus entered Kaliningrad and established the epidemic. Given this possibility as well as relative long period of asymptomatic infection, our early T_mrca_ estimations for CRF03_AB clade (a 5–6 years before the first cases of CRF03_AB detection) do not seem surprising. Apparently, this recombinant was circulating in the region several years before its explosive spread among IDUs; previously, such a scenario has already been demonstrated for the HIV-1 A6 epidemic [18]. Unfortunately, with the data available, it is not possible to verify this hypothesis. Besides the northwest FSU territories, we have also found the migration movements of HIV-1 CRF03_AB recombinant from Kaliningrad to other Russian regions (Perm’, Ekaterinburg, Krasnodar etc.; Fig 3) as well as Central Asian countries (Kazakhstan and Tajikistan) which confirms its role as a local center of this recombinant spread within FSU. The scarcity of epidemiological data (regarding risk factors and human migration flows) made it impossible to clearly determine the driving-force behind the current CRF03_AB dissemination, but almost uncontrolled people migration between FSU countries and heterosexual contacts can greatly contribute to it. Hence, effective strategies aimed at the heterosexual active population, including the young people, are needed to reduce the future burden of HIV-infection.

Some comments should be made concerning the limitations of this study. First, some studies identified subtypes by a single HIV-1 genome region, which may lead to subtyping errors, thus affecting the estimated CRF03_AB prevalence. Second, the small sample size in some countries might not be a representative for the real HIV population. These circumstances combined with the large sequence number variability in each country may in particular, be a bias to the assessment of proposed migration routes and prevent the detection of all viral migration movements in the FSU. Another limitation of our review may be the inclusion of only published studies, which may be a potential source of publication bias. Thus, caution is advised regarding the interpretation of the findings from present study.

## Conclusion

In conclusion, this study supports the initial spread of HIV-1 CRF03_AB recombinant in Kaliningrad city via single viral strain entry followed by expansion to most FSU countries where it prevails unevenly. Our study provides a quantitative summary for this prevalence. Taken together, the findings in the present study contribute to the fundamental understanding of potential CRF-specific differences in the HIV-1 patterns of the epidemic dynamics in FSU, assessment of the social and biological driving forces behind the development of the epidemic process, as well as contribute to prediction of future trends of HIV infections and suggesting effective preventive measures.

## Supporting information

S1 Fig Flowchart of the selection of studies for review. (PDF)

S2 Fig Root-to-tip regression analysis of the phylogenetic temporal signal in HIV-1 CRF03_AB recombinant dataset. (PDF)

S3 Fig Forest plot (A) and meta-regression (B) of HIV-1 CRF03_AB recombinant prevalence in the Russian population.
(PDF)

S4 Fig Bayesian maximum clade credibility phylogeographic tree of the HIV-1 CRF03_AB recombinant. (PDF)

S5 Fig Maximum-likelihood tree of 151 HIV-1 CRF03_AB recombinant *pol* sequences from former Soviet Union and neighboring states. (PDF)

S1 Table Characteristics of studies included in the systematic review and meta-analysis. (DOCX)

S2 Table The number of HIV-1 CRF03_AB sequences from the Los Alamos HIV database for a specific gene or region (data available as of January 25, 2020). (DOCX)

S3 Table GenBank Accession numbers, origin information and sampling date for the HIV-1 sequences used in this study. (XLSX)

S4 Table Best-fit model selection by Bayes Factors for the Bayesian evolutionary analysis. (DOCX)

S1 Checklist PRISMA 2009 checklist. (PDF)
