# Correction: Prevalence and spatiotemporal dynamics of HIV-1 Circulating Recombinant Form 03_AB (CRF03_AB) in the Former Soviet Union countries

**DOI:** 10.1371/journal.pone.0247611

**Published:** 2021-02-19

**Authors:** Aleksey Lebedev, Oksana Pasechnik, Ekaterina Ozhmegova, Anastasiia Antonova, Aleksey Blokh, Liliya Grezina, Tatiana Sandyreva, Natalia Dementeva, Elena Kazennova, Marina Bobkova

In the ‘Results’ subsection of the Abstract, there is an error in the first sentence. The correct sentence is: Our meta-analysis showed that the pooled prevalence of CRF03_AB infection in northwestern FSU region was 9.9% [95%CI: 6.9–12.8].
