# Supplementary figures and images for: Prevalence and spatiotemporal dynamics of HIV-1 Circulating Recombinant Form 03_AB (CRF03_AB) in the Former Soviet Union countries
Source: PLoS One. 2020 Oct 23;15(10):e0241269. doi: 10.1371/journal.pone.0241269 (PMC7584246; doi:10.1371/journal.pone.0241269)

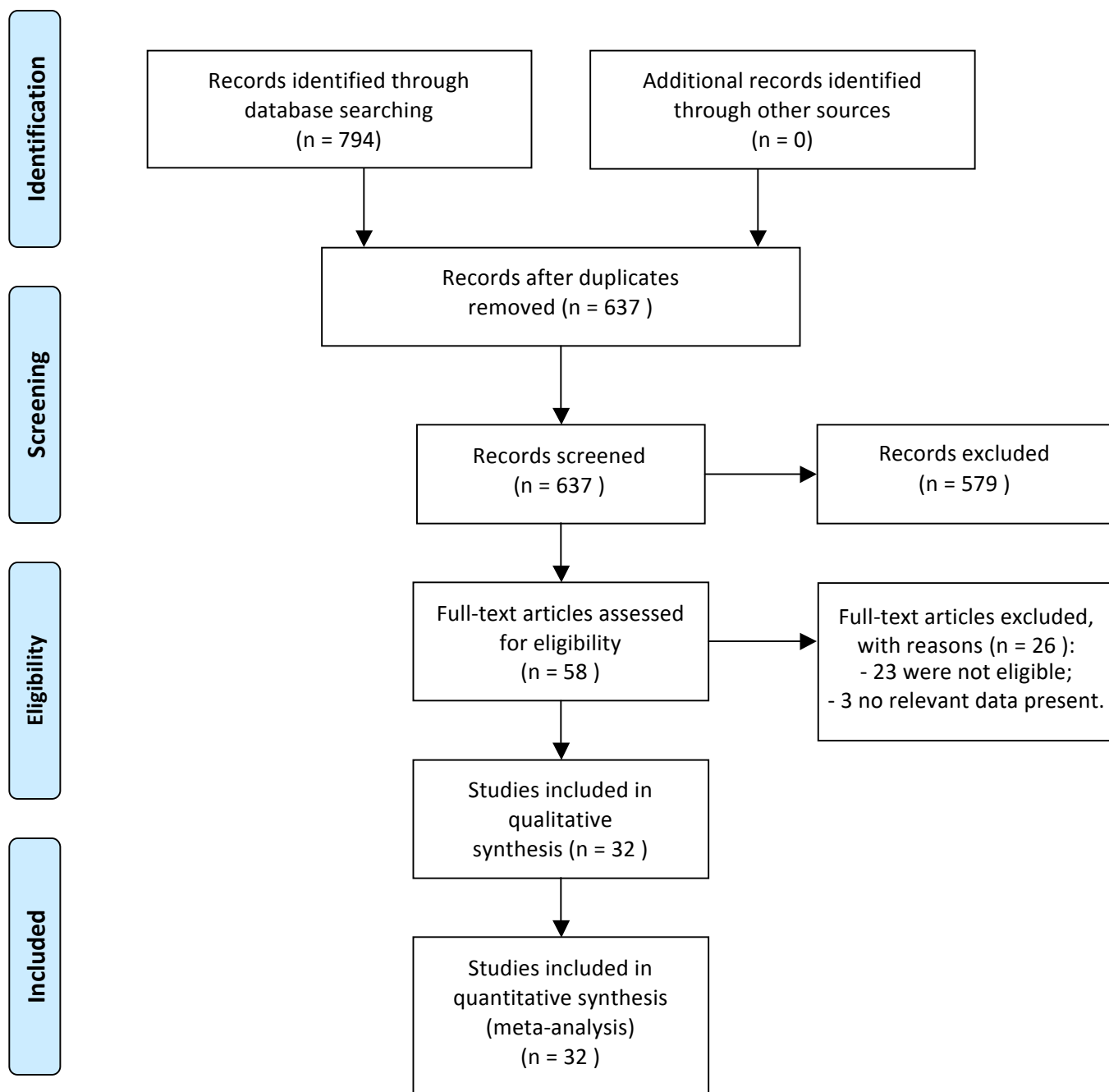

**S1 Fig. Flowchart of the selection of studies for review (PRISMA Diagram, 2009).**

Supplement: S1 Fig — (PDF) [file pone.0241269.s001.pdf]
