# Supplementary material for: Prevalence and spatiotemporal dynamics of HIV-1 Circulating Recombinant Form 03_AB (CRF03_AB) in the Former Soviet Union countries
Source: PLoS One. 2020 Oct 23;15(10):e0241269. doi: 10.1371/journal.pone.0241269 (PMC7584246; doi:10.1371/journal.pone.0241269)
Supplement: S2 Fig — (PDF) [file pone.0241269.s002.pdf]

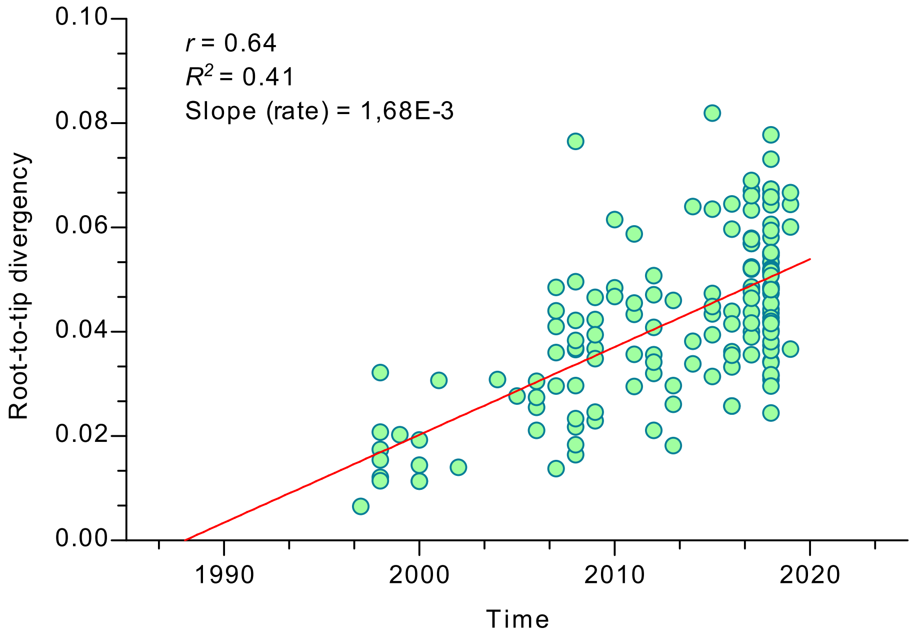

S2 Fig. Root-to-tip regression analysis of the phylogenetic temporal signal in HIV-1 CRF03_AB recombinant dataset. Plot of the root-to-tip genetic distance against sampling time are shown for ML phylogeny estimated from alignment 151 *pol* sequences, sampled between 1997 and 2019. The correlation coefficient was estimated by TempEst v1.5.
