# Supplementary material for: Prevalence and spatiotemporal dynamics of HIV-1 Circulating Recombinant Form 03_AB (CRF03_AB) in the Former Soviet Union countries
Source: PLoS One. 2020 Oct 23;15(10):e0241269. doi: 10.1371/journal.pone.0241269 (PMC7584246; doi:10.1371/journal.pone.0241269)
Supplement: S3 Fig — Forest plot (A) and meta-regression (B) of HIV-1 CRF03_AB recombinant prevalence in the Russian population. (PDF) [file pone.0241269.s003.pdf]

A

Study, year [reference*]	Prevalence, % [95% CI]	CRF03_AB / Total
Liitsola K, 1998 [1]	98.0 [94.1; 101.9]	49 / 50
Smolskaya T, 2006 [2]	57.1 [31.2; 83.1]	8 / 14
Smolskaya T, 2006 [2]	1.2 [-2.0; 4.4]	0 / 42
Smolskaya T, 2006 [2]	1.6 [-2.8; 6.0]	0 / 30
Smolskaya T, 2006 [2]	6.2 [-10.5; 23.0]	0 / 7
Smolskaya T, 2006 [2]	2.5 [-4.3; 9.3]	0 / 19
Smolskaya T, 2006 [2]	5.6 [-9.4; 20.5]	0 / 8
Smolskaya T, 2006 [2]	1.6 [-2.8; 6.0]	0 / 30
Kazennova E, 2008 [3]	76.6 [64.5; 88.7]	36 / 47
Belyakov N, 2012 [4]	0.5 [0.1; 0.9]	5 / 1055
Musatov V, 2013 [5]	0.7 [-1.3; 2.7]	0 / 68
Churina M, 2017 [6]	4.0 [-3.7; 11.7]	1 / 25
Kazennova E, 2017 [7]	1.5 [-1.4; 4.5]	1 / 66
Kazennova E, 2017 [7]	0.7 [-1.3; 2.8]	0 / 66
Ostankova Y, 2019 [8]	1.3 [-1.2; 3.7]	1 / 79
Ozhmegova E, 2020 [9]	32.5 [22.2; 42.8]	26 / 80
Northwestern FD		
(I^2=99.4%; P<0.01)	9.9 [6.9; 12.8]	127 / 1686
Kazachinskaya AG, 2013 [10]	0.2 [-0.4; 0.8]	0 / 250
Kazachinskaya AG, 2013 [10]	1.9 [-3.4; 7.2]	0 / 25
Kazachinskaya AG, 2013 [10]	1.7 [-2.9; 6.2]	0 / 29
Kazennova EV, 2013 [11]	0.5 [-0.9; 1.9]	0 / 99
Kazennova EV, 2013 [11]	5.6 [-9.4; 20.5]	0 / 8
Kazennova EV, 2013 [11]	0.7 [-1.3; 2.7]	0 /69
Gashnikova NM, 2015 [12]	0.8 [-1.4; 3]	0 / 61
Ponomareva OA, 2016 [13]	0.8 [-1.3; 2.8]	0 / 65
Gashnikova NM, 2017 [14]	0.9 [-1.6; 3.5]	0 /53
Neshumaev D, 2019 [15]	0.2 [-0.3; 0.7]	0 / 281
Siberian FD		
(I^2=0%; P=0.98)	0.3 [-0.1; 0.6]	0 / 940
Kazennova EV, 2013 [16]	4,3 [-4; 12,7]	1 / 23
Kazennova EV, 2013 [16]	1,1 [-1,9; 4,1]	0 / 45
Kazennova EV, 2013 [16]	1,4 [-2,5; 5,4]	0 / 34
Kazennova EV, 2014 [17]	1,2 [-2,1; 4,6]	0 / 40
Kazennova EV, 2014 [17]	0,6 [-1; 2,1]	0 / 88
Elisseva VS, 2015 [18]	0,9 [-1,6; 3,5]	0 / 53
Kotova VO, 2016 [19]	0,7 [-0,7; 2,1]	1 / 140
Tumanov AS, 2017 [21]	0,9 [-1,6; 3,5]	0 / 53
Kotova VO, 2018 [20]	0,9 [-1,5; 3,3]	0 / 56
Far Eastern FD		
(I^2=0%; P=0.99)	0.8 [0.1; 1.6]	2 / 532
Kazennova EV, 2013 [24]	15.8 [-0.6; 32.2]	3 / 19
Kazennova EV, 2013 [24]	6.2 [-1.3; 4.1]	8 / 130
Gashnikova NM, 2016 [25]	1.4 [-0.5; 9.7]	1 / 72
Ural FD		
(I^2=66.3%; P=0.05)	4.6 [-0.5; 9.7]	12 / 221
Kazennova EV, 2015 [26]	1.3 [-0.2; 2.7]	3 / 236
Moskaleychik FF, 2015 [22]	4.5 [7.8; 16.9]	0 / 10
Volga FD		
(I^2=0%; P=0.60)	1.3 [-0.1; 2.7]	3 / 246
Moskaleychik FF, 2015 [22]	3.6 [-6.1; 13.3]	0 / 13
Lebedev A, 2019 [23]	0.2 [-0.1; 0.5]	2 / 896
Central FD		
(I^2=0%; P=0.50)	0.2 [-0.1; 0.5]	2 / 909
Overall		
(I^2=98.6%; P<0.01)	**5.9 [4.1; 7.8]**	**146 / 4534**

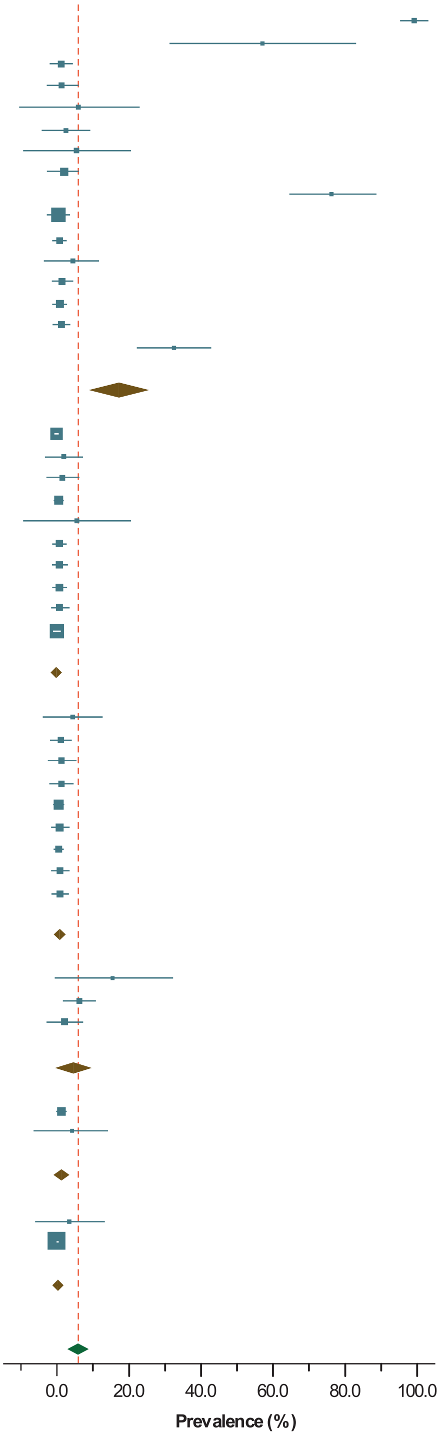

S3 Fig. Forest plot (A) and meta-regression (B) of HIV-1 CRF03_AB recombinant prevalence in the Russian population.

B

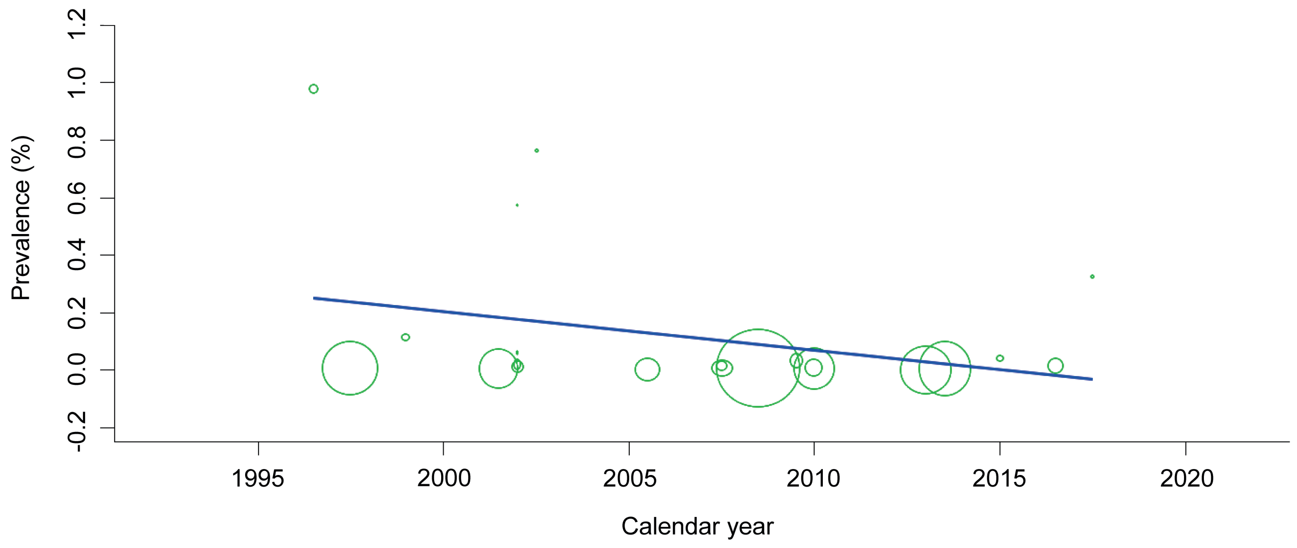

S3 Fig. Continued.
