# Supplementary material for: Prevalence and spatiotemporal dynamics of HIV-1 Circulating Recombinant Form 03_AB (CRF03_AB) in the Former Soviet Union countries
Source: PLoS One. 2020 Oct 23;15(10):e0241269. doi: 10.1371/journal.pone.0241269 (PMC7584246; doi:10.1371/journal.pone.0241269)
Supplement: S4 Fig — (PDF) [file pone.0241269.s004.pdf]

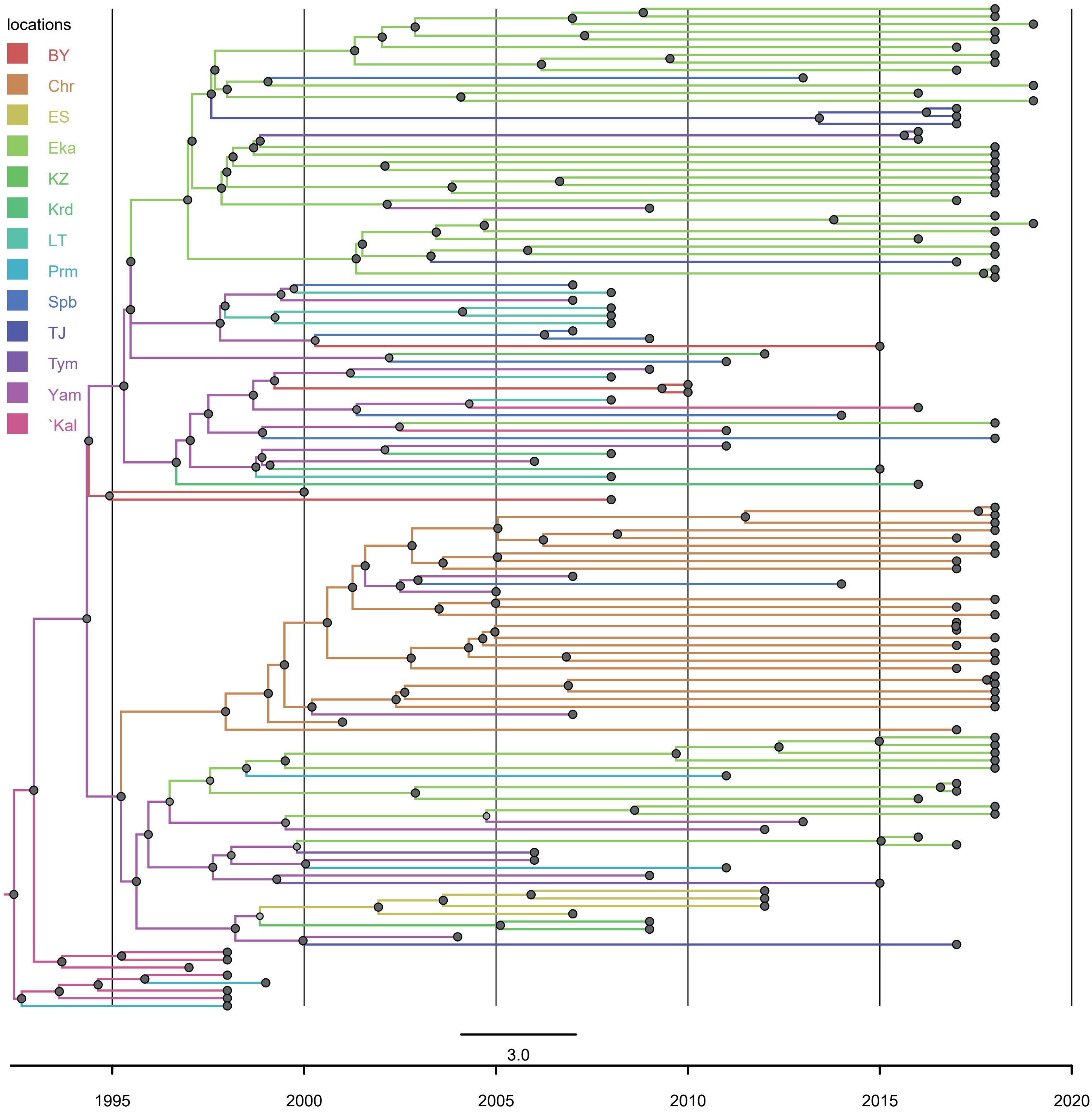

S4 Fig. Bayesian maximum clade credibility phylogeographic tree of the HIV-1 CRF03_AB recombinant. Branches are colored according to the geographical origin for sequence and the most probable ancestral location for clade, as indicated in the legend. The probability location values on nodes are mapped circles using a color gradient/size (greater value, darker grey/greater circle). Belarus, BY; Lithuania, LT; Kazakhstan, KZ; Spain, ES; Tajikistan, TJ; Cherepovets, Chr; Ekaterinburg, Eka; Kaliningrad, Kal; Krasnodar, Krs; Perm`, Prm; Saint-Petersburg, Spb; Tyumen`, Tym; Yamalo-Nenets Autonomous district, Yam.
