# Supplementary material for: Prevalence and spatiotemporal dynamics of HIV-1 Circulating Recombinant Form 03_AB (CRF03_AB) in the Former Soviet Union countries
Source: PLoS One. 2020 Oct 23;15(10):e0241269. doi: 10.1371/journal.pone.0241269 (PMC7584246; doi:10.1371/journal.pone.0241269)
Supplement: S5 Fig — (PDF) [file pone.0241269.s005.pdf]

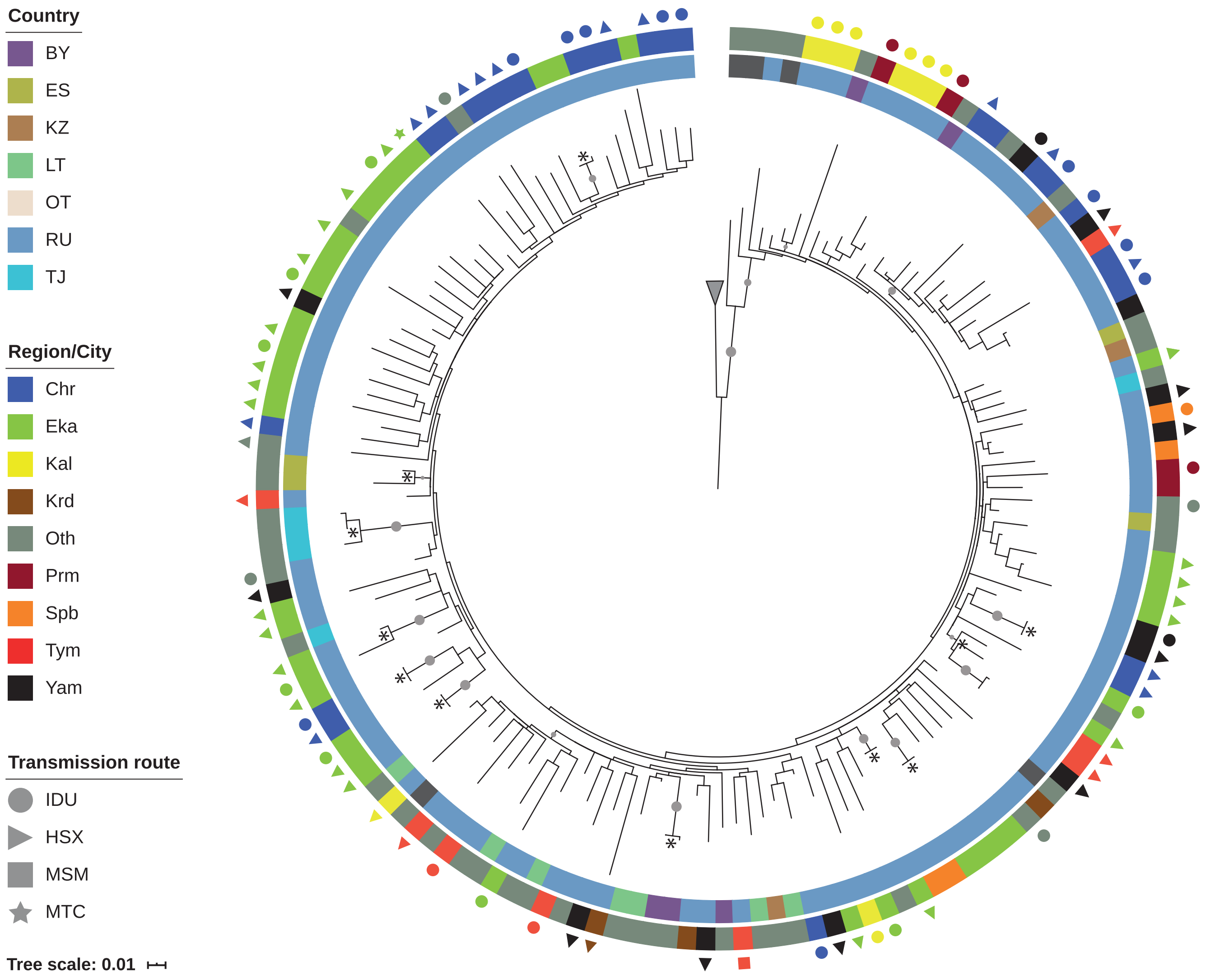

S5 Fig. Maximum-likelihood tree of 151 HIV-1 CRF03_AB recombinant *pol* sequences from former Soviet Union and neighboring states. The reliable clusters (by ClusterPicker) are indicated by an asterisk. The rest designations as on the Fig 3.
