# Supplementary material for: Prevalence and spatiotemporal dynamics of HIV-1 Circulating Recombinant Form 03_AB (CRF03_AB) in the Former Soviet Union countries
Source: PLoS One. 2020 Oct 23;15(10):e0241269. doi: 10.1371/journal.pone.0241269 (PMC7584246; doi:10.1371/journal.pone.0241269)
Supplement: S1 Table — (DOCX) [file pone.0241269.s006.docx]

| **S1 Table. Characteristics of studies included in the systematic review and meta-analysis.** | | | | | | | | |
| --- | --- | --- | --- | --- | --- | --- | --- | --- |
| Author | Date | | | | Material collection area | Number of examined patients | Recombinant forms CRF03_AB detected | Source  link |
|  | Publication | | Material collection | |  |  |  |  |
| **Northwestern Federal District (Russia)** | | | | | | | | |
| Liitsola K. | | 1998 | | 1997-1998 | Kaliningrad | 14 | 8 | [1] |
| Smolskaya T (1) | | 2006 | | 2002 | Vologda District | 14 | 8 | [2] |
| Smolskaya T (2) | | 2006 | | 2002 | Saint-Petersburg | 42 | 0 | [2] |
| Smolskaya T (3) | | 2006 | | 2002 | Pskov | 30 | 0 | [2] |
| Smolskaya T (4) | | 2006 | | 2002 | Republic of Karelia | 7 | 0 | [2] |
| Smolskaya T (5) | | 2006 | | 2002 | Novgorod region | 19 | 0 | [2] |
| Smolskaya T (6) | | 2006 | | 2002 | Arkhangelsk | 8 | 0 | [2] |
| Smolskaya T (7) | | 2006 | | 2002 | Leningrad region | 30 | 0 | [2] |
| Kazennova EV | | 2008 | | 1996-2006 | Vologda District,  Cherepovets | 47 | 36 | [3] |
| Belyakov NA | | 2012 | | 2006-2011 | Saint-Petersburg | 1055 | 5 | [4] |
| Musatov BV | | 2013 | | 2009-2011 | Saint-Petersburg | 68 | 0 | [5] |
| Churina MA | | 2017 | | 2014-2016 | Veliky Novgorod | 25 | 1 | [6] |
| Kazennova EV (1) | | 2017 | | 2001-2014 | Arkhangelsk | 66 | 1 | [7] |
| Kazennova EV (2) | | 2017 | | 2001-2014 | Murmansk | 66 | 0 | [7] |
| Ostankova YV | | 2019 | | 2014-2019 | Arkhangelsk District | 79 | 1 | [8] |
| Ozhmegova EN | | 2020 | | 2018 | Vologda District | 80 | 26 | [9] |
| **Siberian Federal District (Russia)** | | | | | | | | |
| Kazachinskaya AG (1) | | 2013 | | 2010-2013 | Novosibirsk | 250 | 0 | [10] |
| Kazachinskaya AG (2) | | 2013 | | 2010-2013 | Chita | 25 | 0 | [10] |
| Kazachinskaya AG (3) | | 2013 | | 2010-2013 | Novokuznetsk | 29 | 0 | [10] |
| Kazennova EV (1) | | 2013 | | 2005 | Altai region | 69 | 0 | [11] |
| Kazennova EV (2) | | 2013 | | 2005-2009 | Krasnoyarsk region | 99 | 0 | [11] |
| Kazennova EV (3) | | 2013 | | 2006-2007 | Tyva Republic | 8 | 0 | [11] |
| Gashnikova NM | | 2015 | | 2013 | Tomsk | 61 | 0 | [12] |
| Ponamareva OA | | 2016 | | 2014-2016 | Irkutsk region | 65 | 0 | [13] |
| Gashnikova NM | | 2017 | | 2015 | Kemerovo region | 53 | 0 | [14] |
| Neshumaev D | | 2019 | | 2011-2016 | Krasnoyarsk region | 281 | 0 | [15] |
| **Far Eastern Federal District (Russia)** | | | | | | | | |
| Kazennova EV (1) | | 2013 | | 2005 | Magadan region | 23 | 1 | [16] |
| Kazennova EV (2) | | 2013 | | 1998-2007 | Republic of Sakha (Yakutia) | 45 | 0 | [16] |
| Kazennova EV (3) | | 2013 | | 2005 | Khabarovsk region | 34 | 0 | [16] |
| Kazennova EV (1`) | | 2014 | | 2002-2012 | Blagoveshchensk | 40 | 0 | [17] |
| Kazennova EV (2`) | | 2014 | | 2002-2012 | Khabarovsk | 88 | 0 | [17] |
| Elisseva VS | | 2015 | | 2013-2014 | Vladivostok | 53 | 0 | [18] |
| Kotova VO | | 2016 | | 2013-2015 | Far Easten Federal District | 140 | 1 | [19] |
| Kotova VO | | 2018 | | 2015-2017 | Jewish Autonomous Region | 56 | 0 | [20] |
| Tumanov AS | | 2017 | | 2013 | Sakhalin region | 53 | 0 | [21] |
| **Central Federal District (Russia)** | | | | | | | | |
| Moskaleychik FF | | 2015 | | 2008 | Moscow | 13 | 0 | [22] |
| Lebedev A | | 2019 | | 2011-2016 | Moscow region | 896 | 2 | [23] |
| **Ural federal district (Russia)** | | | | | | | | |
| Kazennova EV (1) | | 2013 | | 2006 | Kurgan region | 19 | 3 | [24] |
| Kazennova EV (2) | | 2013 | | 1995-2010 | Yamalo-Nenets Autonomous district | 130 | 8 | [24] |
| Gashnikova NM | | 2016 | | 2000-2015 | Tyumen' | 72 | 1 | [25] |
| **Volga Federal District (Russia)** | | | | | | | | |
| Moskaleychik FF | | 2015 | | 2011 | Perm' | 10 | 0 | [22] |
| Kazennova EV | | 2015 | | 2011-2013 | Volga Federal District | 236 | 3 | [26] |
| **Countries of the East European Region** | | | | | | | | |
| Saad MD | | 2006 | | 2001-2002 | Ukraine | 163 | 0 | [27] |
| Avi R | | 2009 | | 2005-2006 | Estonia | 95 | 0 | [28] |
| Balode D | | 2010 | | 1990-2005 | Latvia | 315 | 1 | [29] |
| Yeryomin VF | | 2012 | | 2008-2011 | Belarus | 139 | 4 | [30] |
| Caplinskas S | | 2013 | | 1990-2008 | Lithuania | 138 | 16 | [31] |
| Avi R | | 2014 | | 2010 | Estonia | 244 | 1 | [32] |
| Vasylyeva TI | | 2018 | | 2012-2015 | Ukraine | 448 | 2 | [33] |
| Huik K | | 2019 | | 2013 | Estonia | 209 | 0 | [34] |
| The numbers of the source link correspond to the list below. | | | | | | | | |

**Source link list**

1. Liitsola K, Tashkinova I, Laukkanen T, Korovina G, Smolskaja T, et al. (1998) HIV-1 genetic subtype A/B recombinant strain causing an explosive epidemic in injecting drug users in Kaliningrad. AIDS 12: 1907-1919.

2. Smolskaya T, Liitsola K, Zetterberg V, Golovanova E, Kevlova N, et al. (2006) HIV epidemiology in the Northwestern Federal District of Russia: dominance of HIV type 1 subtype A. AIDS Res Hum Retroviruses 22: 1074-1080.

3. Kazennova EV, Bronnikova AV, Kuzin SN, Kirillova IL, Ershova ON, et al. (2008) [Molecular genetic characteristics of HIV-1 variants circulating in Cherepovets, Vologda region: the second case of the epidemic outbreak caused by the recombinant gagAenvB]. Vopr Virusol 53: 23-27.

4. Belyakov NA, Rosental VV, Dementeva NY, Vinogradova TM, Sizova NV (2012) [Mathematical modelling and general trends of circulation of HIV subtypes and recombinant forms]. HIV Infect Immun Dis 2: 7-18.

5. Musatov VB, Yakovlev AA, Tirgina TV, Ladnaya NN (2013) [The prognostic significance of the results of genotyping human immunodeficiency virus isolated from the patients with primary HIV-infection in 2009 and 2011 in St. Petersburg]. Vest St. Petersburg Univ 11: 171-178.

6. Churina MA, Ostankova YV, Semenov AV, Nikitina NA, Rosolovsky AP, et al. (2017) [HIV-1 drug-resistance and molecular epidemiology in patients with art failure in Veliky Novgorod]. HIV Infect Immun Dis 1: 82-92.

7. Kazennova EV, Laga VY, Gromov KB, Sankov MN, Popova ES, et al. (2017) [Molecular epidemiological analysis of HIV infection in northern seaports of Russia]. Vopr Virusol 62: 154-161.

8. Ostankova YV, Schemelev AN, Zueva EV, Churina MA, Valutite DE, et al. (2019) [HIV molecular epidemiology and pharmaco-resistance in patients with antiretroviral therapy failure in Arkhangelsk District]. HIV Infect Immun Dis 4: 79-90.

9. Ozhmegova EN, Antonova AA, Lebedev AV, Melnikova TN, Krylova TV, et al. (2020) [Genetic profile of HIV-1 in the Vologda region: domination of CRF03_AB and rapid distribution of URFs]. HIV Infect Immun Dis 2: 79-88.

10. Kazachinskaya АG, Bogachev VV, Baryshev PB, Tommenin АV, Chybareva ЕА, et al. (2013) [Territorial features of spread of HIV-1 genetic options in Siberian federal district]. Medicine and Education in Siberia 3: 12.

11. Kazennova EV, Vasil'ev AV, Lapovok IA, Grishechkin AE, Laga V, et al. (2013) [HIV-1 genetic variants in the Asian part of Russia: a study (2005-2010)]. Vopr Virusol 58: 28-35.

12. Gashnikova NM, Bogachev VV, Baryshev PB, Totmenin AV, Gashnikova MP, et al. (2015) [A rapid expansion of HIV-1 CRF63_02A1 among newly diagnosed HIV-infected individuals in the Tomsk Region, Russia]. AIDS Res Hum Retroviruses 31: 456-460.

13. Ponomareva OA, Revizor AO, Kruglova EA, Plotnikova YK, Naumova ES (2016) [Genetic diversity of HIV-1 in Irkutsk region]. Lab Serv 1: 33-37

14. Gashnikova NM, Zyryanova DP, Astakhova EM, Ivlev VV, Gashnikova MP, et al. (2017) Predominance of CRF63_02A1 and multiple patterns of unique recombinant forms of CRF63_A1 among individuals with newly diagnosed HIV-1 infection in Kemerovo Oblast, Russia. Arch Virol 162: 379-390.

15. Neshumaev D, Lebedev A, Malysheva M, Boyko A, Skudarnov S, et al. (2019) Molecular Surveillance of HIV-1 Infection in Krasnoyarsk Region, Russia: Epidemiology, Phylodynamics and Phylogeography. Curr HIV Res 17: 114-125.

16. Kazennova EV, Vasil'ev AV, Lapovok IA, Grishechkin AE, Laga V, et al. (2013) [HIV-1 genetic variants in the Asian part of Russia: a study (2005-2010)]. Vopr Virusol 58: 28-35.

17. Kazennova EV, Neshumaev DA, Rukavitsin DV, Lapovok IA, Laga V, et al. (2014) [Molecular epidemiological analysis of the HIV infection in the Blagoveshchensk and Khabarovsk Area (Russian Far East)]. Vopr Virusol 59: 31-36.

18. Eliseeva VS, Kruglyak SP, Sklyar LF, Smahno E (2015) [Prevalence of HIV-1 drug resistance mutations in Primorsky Region]. HIV Infect Immun Dis 7: 49-54.

19. Kotova VO, Balakhontseva LA, Trotsenko OE (2016) [Analysis of HIV-1 drug resistance in regions of the Far Easten Federal district]. HIV Infect Immun Dis 3:53-58.

20. Kotova VO, Trotsenko OE, Balakhontseva LA, Bazykina EA, Yanovich OA, et al. (2018) [Molecular-epidemiological characteristics of Hiv-1 variants circulating in the Jewish Autonomous Region territory]. HIV Infect Immun Dis 4: 90-99.

21. Tumanov AS, Kazennova EV, Gromov KB, Lomakina EA, Zozylya EY, , et al. (2018) [The molecular epidemiological analysis of HIV infection in Sakhalin region, Russia]. HIV Infect Immun Dis 3: 113-120.

22. Moskaleychik FF, Laga VY, Delgado E, Vega Y, Fernandez-Garcia A, et al. (2015) [Rapid spread of the HIV-1 circular recombinant CRF02-AG in Russia and neighboring countries]. Vopr Virusol 60: 14-19.

23. Lebedev A, Lebedeva N, Moskaleychik F, Pronin A, Kazennova E, et al. (2019) Human Immunodeficiency Virus-1 Diversity in the Moscow Region, Russia: Phylodynamics of the Most Common Subtypes. Front Microbiol 10: 320.

24. Kazennova EV, Vasil'ev AV, Lapovok IA, Grishechkin AE, Laga V, et al. (2013) [HIV-1 genetic variants in the Asian part of Russia: a study (2005-2010)]. Vopr Virusol 58: 28-35..

25. Gashnikova NM, Astakhova EM, Gashnikova MP, Bocharov EF, Petrova SV, et al. (2016) HIV-1 Epidemiology, Genetic Diversity, and Primary Drug Resistance in the Tyumen Oblast, Russia. Biomed Res Int 2016: 2496280.

26. Kazennova EV, Lapovok IA, Lebedev AV, Laga VY, Glushchenko NV, et al. (2015) [Analysis of HIV drugs resistance in Privolzhskiy federal district of the Russian Federation]. HIV Infect Immun Dis 2: 7-18.

27. Saad MD, Aliev Q, Botros BA, Carr JK, Gomatos PJ, et al. (2006) Genetic forms of HIV Type 1 in the former Soviet Union dominate the epidemic in Azerbaijan. AIDS Res Hum Retroviruses 22: 796-800.

28. Avi R, Huik K, Sadam M, Karki T, Krispin T, et al. (2009) Absence of genotypic drug resistance and presence of several naturally occurring polymorphisms of human immunodeficiency virus-1 CRF06_cpx in treatment-naive patients in Estonia. J Med Virol 81: 953-958.

29. Balode D, Ferdats A, Dievberna I, Viksna L, Rozentale B, et al. (2004) Rapid epidemic spread of HIV type 1 subtype A1 among intravenous drug users in Latvia and slower spread of subtype B among other risk groups. AIDS Res Hum Retroviruses 20: 245-249.

30. Yeryomin VF, Gasich EL, Sosinovich SV, Suyetnov ON, Grushko PN, et al. (2012) [Molecular epidemiology of HIV/AIDS in Belarus (2008—2011)]. Zdrav 1: 25-34.

31. Caplinskas S, Loukachov VV, Gasich EL, Gilyazova AV, Caplinskiene I, et al. (2013) Distinct HIV type 1 strains in different risk groups and the absence of new infections by drug-resistant strains in Lithuania. AIDS Res Hum Retroviruses 29: 732-737.

32. Avi R, Huik K, Pauskar M, Ustina V, Karki T, et al. (2014) Transmitted drug resistance is still low in newly diagnosed human immunodeficiency virus type 1 CRF06_cpx-infected patients in Estonia in 2010. AIDS Res Hum Retroviruses 30: 278-283.

33. Vasylyeva TI, Liulchuk M, Friedman SR, Sazonova I, Faria NR, et al. (2018) Molecular epidemiology reveals the role of war in the spread of HIV in Ukraine. Proc Natl Acad Sci USA 115: 1051-1056.

34. Soodla P, Huik K, Pauskar M, Cuypers L, Van Laethem K, et al. (2019) Stable level of HIV transmitted drug resistance in Estonia despite significant scale-up of antiretroviral therapy. Infect Genet Evol 75: 103901.
