# Supplementary material for: Prevalence and spatiotemporal dynamics of HIV-1 Circulating Recombinant Form 03_AB (CRF03_AB) in the Former Soviet Union countries
Source: PLoS One. 2020 Oct 23;15(10):e0241269. doi: 10.1371/journal.pone.0241269 (PMC7584246; doi:10.1371/journal.pone.0241269)
Supplement: S2 Table — (DOCX) [file pone.0241269.s007.docx]

| **S2 Table. The number of HIV-1 CRF03_AB sequences from the Los Alamos HIV database for a specific gene or region.** | | | | | | | | | | | | | | | | | | | | | | | |
| --- | --- | --- | --- | --- | --- | --- | --- | --- | --- | --- | --- | --- | --- | --- | --- | --- | --- | --- | --- | --- | --- | --- | --- |
| **#** | **Genome region** | **All** | **Countries** | | | | | | | | | | | | | | | | | | | | |
|  |  |  | **Afganistan** | **Azerbaijan** | **Belgium** | **Belarus** | **China** | **Germany** | **Estonia** | **Spain** | **United**  **kingdom** | **Italy** | **Japan** | **Kyrgyzstan** | **Kazahstan** | **Lithuania** | **Luxembourg** | **Russian**  **Federation** | **Seychelles** | **Sweden** | **Tajikistan** | **Ukraine** | **Uzbekistan** |
| 1 | near Full length | 4 |  |  |  | 1 |  |  |  |  | 1 |  |  |  |  |  |  | 2 |  |  |  |  |  |
| 2 | LTR | 4 |  |  |  | 1 |  |  |  |  | 1 |  |  |  |  |  |  | 2 |  |  |  |  |  |
| 3 | p17 | 102 |  |  |  | 19 |  |  |  |  | 1 |  | 2 |  |  | 76 |  | 2 |  | 2 |  |  |  |
| 4 | p24 (5'-segment) | 148 | 1 |  |  | 19 |  |  |  |  | 1 |  |  |  |  | 75 |  | 49 |  | 2 |  | 1 |  |
| 5 | p17-p24 (#3∩#4) | 24 |  |  |  | 19 |  |  |  |  | 1 |  |  |  |  |  |  | 2 |  | 2 |  |  |  |
| 6 | p7 | 65 | 1 |  |  | 7 |  |  | 3 |  | 1 |  |  |  |  | 1 |  | 51 |  |  |  | 1 |  |
| 7 | p6 | 112 |  | 1 | 9 | 7 | 1 | 1 | 4 | 7 | 1 |  |  | 1 | 1 | 7 | 2 | 63 | 1 |  | 5 | 1 |  |
| 8 | p7-p6 (#6∩#7) | 15 |  |  |  | 7 |  |  | 3 |  | 1 |  |  |  |  |  |  | 4 |  |  |  |  |  |
| 9 | protease (PR) | 112 |  | 1 | 9 | 7 | 1 | 1 | 3 | 7 | 1 |  |  | 1 | 1 | 7 | 2 | 64 | 1 |  | 5 | 1 |  |
| 10 | reverse transcriptase (RT) | 96 |  | 1 | 1 | 7 | 1 |  | 1 | 4 | 1 |  |  | 1 | 3 | 7 |  | 63 |  |  | 5 | 1 |  |
| 11 | PR-RT (#9∩#10) | 92 |  | 1 |  | 7 | 1 |  | 1 | 4 | 1 |  |  |  | 1 | 7 |  | 63 |  |  | 5 | 1 |  |
| 12 | p24-RT (#4∩#9 = #7∩#10) | 4 |  |  |  | 1 |  |  |  |  | 1 |  |  |  |  |  |  | 2 |  |  |  |  |  |
| 13 | integrase (int) | 8 |  |  |  | 1 |  |  |  |  | 1 | 1 |  | 1 | 2 |  |  | 2 |  |  |  |  |  |
| 14 | V3-loop | 169 |  |  |  | 1 |  |  | 1 | 1 | 1 | 2 |  |  |  | 10 |  | 149 |  |  |  | 4 | 4 |
| 15 | V4-loop | 8 |  |  |  | 1 |  |  |  |  | 1 |  |  |  |  |  |  | 2 |  |  |  | 4 |  |
| 16 | V3C3V4 | 11 |  |  |  | 1 |  |  |  |  | 1 |  |  |  |  |  |  | 5 |  |  |  | 4 |  |
| 17 | C3 | 169 |  |  |  | 1 |  |  | 1 | 1 | 1 | 2 |  |  |  | 10 |  | 149 |  |  |  | 4 | 4 |
| 18 | full env | 5 |  |  |  | 1 |  |  |  |  | 1 |  |  |  |  |  |  | 3 |  |  |  |  |  |
| 19 | nef | 5 |  |  |  | 1 |  |  |  |  | 1 |  |  |  |  |  |  | 3 |  |  |  |  |  |
|  | Total ^a^ | 434 | 1 | 1 | 10 | 23 | 1 | 1 | 5 | 8 | 1 | 3 | 2 | 3 | 5 | 93 | 2 | 257 | 1 | 2 | 5 | 6 | 4 |
| Number of sequences for each genomic region was defined manually.  ^a^ Indicates the total number of sequences for each country by LANL HIV Database. Three sequences from Belarus (FR729474,FR729475) and Armenian (KM229395) removed due to discrepancy with the subtype CRF03_AB; eighteen Belarusian sequences (AF413988-AF414006) were multiple data on the same patient (ID19536). | | | | | | | | | | | | | | | | | | | | | | | |
