# Supplementary material for: Prevalence and spatiotemporal dynamics of HIV-1 Circulating Recombinant Form 03_AB (CRF03_AB) in the Former Soviet Union countries
Source: PLoS One. 2020 Oct 23;15(10):e0241269. doi: 10.1371/journal.pone.0241269 (PMC7584246; doi:10.1371/journal.pone.0241269)
Supplement: S4 Table — (DOCX) [file pone.0241269.s009.docx]

| **S4 Table. Best-fit model selection by Bayes Factors (BF) for the Bayesian evolutionary analysis.** | | | | | | | | | |
| --- | --- | --- | --- | --- | --- | --- | --- | --- | --- |
| **#** |  | **1** | **2** | **3** | **4** | **5** | **6** | **7** | **8** |
| 1 | **Strict х Constant size** |  | -9,7 | -6,3 | 2,6 | 45,6 | -45,5 | -44,1 | 49,5 |
| 2 | **Strict х Exponential growth** |  |  | -3,4 | -7,1 | 35,9 | 35,7 | 34,3 | 39,8 |
| 3 | **Strict х Logistic growth** |  |  |  | -3,7 | 39,3 | 39,2 | 37,8 | 43,2 |
| 4 | **Strict х Bayesian Skyline** |  |  |  |  | -43,0 | -42,9 | -41,5 | 47,0 |
| 5 | **Lognormal (relaxed) х Constant size** |  |  |  |  |  | 0,1 | 1,5 | 3,9 |
| 6 | **Lognormal (relaxed) х Exponential growth** |  |  |  |  |  |  | 1,4 | 4,1 |
| 7 | **Lognormal (relaxed) х Logistic growth** |  |  |  |  |  |  |  | 5,5 |
| 8 | **Lognormal (relaxed) х Bayesian Skyline** |  |  |  |  |  |  |  |  |
| Diagonal values represent ln (BF) which is the difference of the marginal likelihood of model combinations. Model comparisons in a Bayesian framework were performed with Tracer v1.5. | | | | | | | | | |
