# Supplementary material for: Prevalence and spatiotemporal dynamics of HIV-1 Circulating Recombinant Form 03_AB (CRF03_AB) in the Former Soviet Union countries
Source: PLoS One. 2020 Oct 23;15(10):e0241269. doi: 10.1371/journal.pone.0241269 (PMC7584246; doi:10.1371/journal.pone.0241269)
Supplement: S1 Checklist — (PDF) [file pone.0241269.s010.pdf]

PRISMA 2009 Checklist

Section/topic	#	Checklist item	Reported on page #
**TITLE**			
Title	1	Identify the report as a systematic review, meta-analysis, or both.	Not reported*
**ABSTRACT**			
Structured summary	2	Provide a structured summary including, as applicable: background; objectives; data sources; study eligibility criteria, participants, and interventions; study appraisal and synthesis methods; results; limitations; conclusions and implications of key findings; systematic review registration number.	2
**INTRODUCTION**			
Rationale	3	Describe the rationale for the review in the context of what is already known.	4-5
Objectives	4	Provide an explicit statement of questions being addressed with reference to participants, interventions, comparisons, outcomes, and study design (PICOS).	5
**METHODS**			
Protocol and registration	5	Indicate if a review protocol exists, if and where it can be accessed (e.g., Web address), and, if available, provide registration information including registration number.	Not reported
Eligibility criteria	6	Specify study characteristics (e.g., PICOS, length of follow-up) and report characteristics (e.g., years considered, language, publication status) used as criteria for eligibility, giving rationale.	26
Information sources	7	Describe all information sources (e.g., databases with dates of coverage, contact with study authors to identify additional studies) in the search and date last searched.	5
Search	8	Present full electronic search strategy for at least one database, including any limits used, such that it could be repeated.	5 (not full)*
Study selection	9	State the process for selecting studies (i.e., screening, eligibility, included in systematic review, and, if applicable, included in the meta-analysis).	5
Data collection process	10	Describe method of data extraction from reports (e.g., piloted forms, independently, in duplicate) and any processes for obtaining and confirming data from investigators.	Not reported*
Data items	11	List and define all variables for which data were sought (e.g., PICOS, funding sources) and any assumptions and simplifications made.	Not reported*
Risk of bias in individual studies	12	Describe methods used for assessing risk of bias of individual studies (including specification of whether this was done at the study or outcome level), and how this information is to be used in any data synthesis.	Not reported
Summary measures	13	State the principal summary measures (e.g., risk ratio, difference in means).	6
Synthesis of results	14	Describe the methods of handling data and combining results of studies, if done, including measures of consistency (e.g., I^2) for each meta-analysis.	5

*** Comments for «PRISMA 2009 Checklist»**

PRISMA 2009 Checklist

Section/topic	#	Checklist item	Reported on page #
Risk of bias across studies	15	Specify any assessment of risk of bias that may affect the cumulative evidence (e.g., publication bias, selective reporting within studies).	Not reported*
Additional analyses	16	Describe methods of additional analyses (e.g., sensitivity or subgroup analyses, meta-regression), if done, indicating which were pre-specified.	6
**RESULTS**			
Study selection	17	Give numbers of studies screened, assessed for eligibility, and included in the review, with reasons for exclusions at each stage, ideally with a flow diagram.	26
Study characteristics	18	For each study, present characteristics for which data were extracted (e.g., study size, PICOS, follow-up period) and provide the citations.	26
Risk of bias within studies	19	Present data on risk of bias of each study and, if available, any outcome level assessment (see item 12).	Not reported
Results of individual studies	20	For all outcomes considered (benefits or harms), present, for each study: (a) simple summary data for each intervention group (b) effect estimates and confidence intervals, ideally with a forest plot.	26
Synthesis of results	21	Present results of each meta-analysis done, including confidence intervals and measures of consistency.	26
Risk of bias across studies	22	Present results of any assessment of risk of bias across studies (see Item 15).	Not reported
Additional analysis	23	Give results of additional analyses, if done (e.g., sensitivity or subgroup analyses, meta-regression [see Item 16]).	26
**DISCUSSION**			
Summary of evidence	24	Summarize the main findings including the strength of evidence for each main outcome; consider their relevance to key groups (e.g., healthcare providers, users, and policy makers).	15
Limitations	25	Discuss limitations at study and outcome level (e.g., risk of bias), and at review-level (e.g., incomplete retrieval of identified research, reporting bias).	Not reported
Conclusions	26	Provide a general interpretation of the results in the context of other evidence, and implications for future research.	15
**FUNDING**			
Funding	27	Describe sources of funding for the systematic review and other support (e.g., supply of data); role of funders for the systematic review.	Not reported

*** Comments for «PRISMA 2009 Checklist»**

Comments for «PRISMA 2009 Checklist»

1. This study included a Systematic Review and Meta-Analysis of the prevalence of CRF03_AB throughout the territory of Russian and northwestern FSU countries. Articles published from 1-Oct-1998 to 10-May-2020 with HIV genetic material collection period with a duration of 29 years (1990 - 2019) are included in this review. Many of them are available only in Russian language publications.

8. We searched for articles published in Russian Science Citation Index and PubMed in for a depth of 22 years with the search terms “subtype”, “HIV”, “recombinant”, and “genotype”. Search strategy in Russian Science Citation Index: (subtype *) & (HIV) & (recombinant *) & (genotype *). Additionally, a search was carried out in Google.Scholar and a manual search in bibliographic lists from the found articles.

10. For Systematic Review, the findings of cross-sectional studies on the estimation the prevalence of CRF03_AB recombinant in Russia and neighboring east European FSU states were selected.

11. Two authors reviewed the retrieved studies independently. After removing the duplicates, the full-texts were obtained for eligible articles and the following information was extracted: name of the first author, year of publication, location of study (territory), material collection dates, sample size, reported prevalence of CRF03_AB. Data on multiple populations from a single original publication were considered separately.

At first, we estimated the pooled CRF03_AB prevalence in Russia, for which all in-Russia studies were divided into 6 subgroups in accordance with the federal districts (Northwestern, Siberian, Far Eastern, Central, Ural and Volga). In meta-regression, which characterized the change of CRF03_AB prevalence in region, the collection dates had been used as regressor.

Then, after identifying the region of Russia with the highest CRF03_AB prevalence (Northwestern federal district), we estimated the pooled CRF03_AB prevalence in northwestern FSU countries, including Northwestern federal district of Russia.

12. Publications that had an unclear description for the study population, location and clinical material collection dates, and those that did not have full text as well as theses and materials of conferences, dissertations, and presentations of scientific reports, were excluded.

15. Objectives have not been evaluated because the study included the findings of cross-sectional studies.
